# Supplementary material for: Multiscale networks in Alzheimer’s disease identify brain hypometabolism as central across biological scales
Source: PLoS Comput Biol. 2025 Oct 17;21(10):e1013583. doi: 10.1371/journal.pcbi.1013583 (PMC12548887; doi:10.1371/journal.pcbi.1013583)
Supplement: S1 Table — (PDF) [file pcbi.1013583.s001.pdf]

Description of the variables in the Genetic dataset

Num	Node	Description
1	APOE_A1	Copy 1 of *APOE* gene. The numbers correspond to their alleles versions: E2, E3, E4 are 2, 3, 4 respectively.
2	APOE_A2	Copy 2 of *APOE* gene
3	APOE	Corresponds to both alleles together: 0 (no E4 allele), 1 (heterozygote, one E4 allele) and 2 (homozygote, both alleles E4)
4	TOMM40_A1	Allele 1 of Translocase of Outer Mitochondria Membrane 40 gene (in length, based on the modes of the distributions of the number of T-residues: Short (S) $T \leq 19$, Long (L) $20 \leq T \leq 29$ and Very Long (VL) $T \geq 30$)
5	TOMM40_A2	Allele 2 of Translocase of Outer Mitochondria Membrane 40 gene (in length, based on the modes of the distributions of the number of T-residues: Short (S) $T \leq 19$, Long (L) $20 \leq T \leq 29$ and Very Long (VL) $T \geq 30$)
6	PHS	Polygenic Hazard Score
7	CIR	Cummulative Incidence Rate
