# Supplementary material for: Multiscale networks in Alzheimer’s disease identify brain hypometabolism as central across biological scales
Source: PLoS Comput Biol. 2025 Oct 17;21(10):e1013583. doi: 10.1371/journal.pcbi.1013583 (PMC12548887; doi:10.1371/journal.pcbi.1013583)
Supplement: S2 Table — (PDF) [file pcbi.1013583.s002.pdf]

Description of the variables in the Molecular dataset

Num	Node	Description
8	EUR_AB42	CSF β-Amyloid 1-42 pg/mL - EUROIMMUN
9	EUR_AB42/40	CSF β-Amyloid 1-42, 1-40 Ratio - EUROIMMUN
10	FUJI_AB42	CSF β-amyloid 1-42 pg/mL - FUJIREBIO
11	FUJI_AB42/40	CSF β-amyloid 1-42, 1-40 Ratio - FUJIREBIO
12	UGOT_PLASMAPTAU	phosphorylated tau longitudinal plasma pg/ml - UGOT
13	TS_RATIO	TL/SCG Telomere length/single copy gene ratio of DNA from blood
15	TL	Telomere Length L in base pairs
16	BACE	CSF Beta Secretase pM - Biomarkers Consortium Project
17	APP	CSF (Cerebrospinal Fluid) Amyloid Precursor Protein pM - Biomarkers Consortium Project
18	UPK_AB42	CSF β-amyloid - UPENN
19	UPK_TAU	CSF Total Tau - UPENN
20	UPK_PTAU	CSF phosphorylated-tau - UPENN
21	UPKelec_AB42	CSF β-amyloid using the fully automated Roche Elecsys immunoassay - UPENN
22	UPKelec_TAU	CSF Total Tau using the fully automated Roche Elecsys immunoassay - UPENN
23	UPKelec_PTAU	CSF phosphorylated-tau using the fully automated Roche Elecsys immunoassay - UPENN
24	UPplasma_AB42	Plasma β-Amyloid 1-42 pg/mL - UPENN

UPENN: University of Pennsylvania, UGOT: University of Gothenburg
