# Supplementary material for: Multiscale networks in Alzheimer’s disease identify brain hypometabolism as central across biological scales
Source: PLoS Comput Biol. 2025 Oct 17;21(10):e1013583. doi: 10.1371/journal.pcbi.1013583 (PMC12548887; doi:10.1371/journal.pcbi.1013583)
Supplement: S3 Table — (PDF) [file pcbi.1013583.s003.pdf]

Description of the variables in the PET dataset

Num	Node	Description
25	MEAN_METAROI_ PONSVERMIS	Glucose metabolism normalized to pons-vermis mean of FDG-PET within ROI (Left Angular Gyrus, Right Angular Gyrus, Bilateral Posterior Cingular, Left Inferior Temporal Gyrus, Right Inferior Temporal Gyrus) - UC Berkeley
26	BRAAK1_SUVR	Weighted AV1451 mean of Braak region I (AV1451 PVC analysis) - UC Berkeley
27	BRAAK34_SUVR	Weighted AV1451 mean of Braak region III and IV (AV1451 PVC analysis) - UC Berkeley
28	BRAAK56_SUVR	Weighted AV1451 mean of Braak region V and VI (AV1451 PVC analysis) - UC Berkeley
29	CEREBELLUMGREYMATTER_ AV45	Reference region - AV45 mean of cerebellar grey matter - UC Berkeley
30	WHOLECEREBELLUM_AV45	Reference region - AV45 mean of whole cerebellum - UC Berkeley
31	FRONTAL_AV45	Weighted AV45 mean in frontal regions - UC Berkeley
32	CINGULATE_AV45	Weighted AV45 mean in anterior/posterior cingulate regions - UC Berkeley
33	PARIETAL_AV45	Weighted AV45 mean in lateral parietal regions - UC Berkeley
34	TEMPORAL_AV45	Weighted AV45 mean in lateral temporal regions - UC Berkeley
35	COMPOSITE_AV45	Weighted AV45 mean in frontal, cingulate, parietal and temporal regions - UC Berkeley
36	SUMMARYSUVR_WHOLE CEREBNORM_AV45	Summary AV45 cortical SUVR normalized by whole cerebellum - UC Berkeley
37	SUMMARYSUVR_COMPOSITE_ REFNORM_AV45	Summary AV45 cortical SUVR normalized by composite ref region - UC Berkeley

38	LH_CAUDALANTERIOR CINGULATE_AV45	SUVr for AV45 in caudal anterior cingulate region of the left hemisphere - UC Berkeley
39	LH_CAUDALMIDDLEFRONTAL_AV45	SUVr for AV45 in caudal middle frontal region of the left hemisphere - UC Berkeley
40	LH_CUNEUS_AV45	SUVr for AV45 in cuneus region of the left hemisphere - UC Berkeley
41	LH_ENTORHINAL_AV45	SUVr for AV45 in entorhinal region of the left hemisphere - UC Berkeley
42	LH_FRONTALPOLE_AV45	SUVr for AV45 in frontal pole region of the left hemisphere - UC Berkeley
43	LH_FUSIFORM_AV45	SUVr for AV45 in frontal pole region of the left hemisphere - UC Berkeley
44	LH_INFERIORPARIETAL_AV45	SUVr for AV45 in inferior parietal region of the left hemisphere - UC Berkeley
45	LH_INFERIORTEMPORAL_AV45	SUVr for AV45 in inferior temporal region of the left hemisphere - UC Berkeley
46	LH_INSULA_AV45	SUVr for AV45 in insula region of the left hemisphere - UC Berkeley
47	LH_ISTHMUSCINGULATE_AV45	SUVr for AV45 in isthmus cingulate region of the left hemisphere - UC Berkeley
48	LH_LATERALOCCIPITAL_AV45	SUVr for AV45 in lateral occipital region of the left hemisphere - UC Berkeley
49	LH_LATERALORBITOFRONTAL_AV45	SUVr for AV45 in lateral orbitofrontal region of the left hemisphere - UC Berkeley
50	LH_LINGUAL_AV45	SUVr for AV45 in lingual region of the left hemisphere - UC Berkeley
51	LH_MEDIALORBITOFRONTAL_AV45	SUVr for AV45 in medial orbitofrontal region of the left hemisphere - UC Berkeley
52	LH_MIDDLETEMPORAL_AV45	SUVr for AV45 in middle temporal region of the left hemisphere - UC Berkeley

53	LH_PARACENTRAL_AV45	SUVr for AV45 in paracentral region of the left hemisphere - UC Berkeley
54	LH_PARAHIPPOCAMPAL_AV45	SUVr for AV45 in parahippocampal region of the left hemisphere - UC Berkeley
55	LH_PARSOPERCULARIS_AV45	SUVr for AV45 in pars opercularis region of the left hemisphere - UC Berkeley
56	LH_PARSORBITALIS_AV45	SUVr for AV45 in pars orbitalis region of the left hemisphere - UC Berkeley
57	LH_PARSTRIANGULARIS_AV45	SUVr for AV45 in pars triangularis region of the left hemisphere - UC Berkeley
58	LH_POSTCENTRAL_AV45	SUVr for AV45 in postcentral region of the left hemisphere - UC Berkeley
59	LH_POSTERIORCINGULATE_AV45	SUVr for AV45 in posterior cingulate region of the left hemisphere - UC Berkeley
60	LH_PRECENTRAL_AV45	SUVr for AV45 in precentral region of the left hemisphere - UC Berkeley
61	LH_PRECUNEUS_AV45	SUVr for AV45 in precuneus region of the left hemisphere - UC Berkeley
62	LH_ROSTRALANTERIOR CINGULATE_AV45	SUVr for AV45 in rostral anterior cingulate region of the left hemisphere - UC Berkeley
63	LH_ROSTRALMIDDLEFRONTAL_AV45	SUVr for AV45 in rostral middle frontal region of the left hemisphere - UC Berkeley
64	LH_SUPERIORFRONTAL_AV45	SUVr for AV45 in superior frontal region of the left hemisphere - UC Berkeley
65	LH_SUPERIORPARIETAL_AV45	SUVr for AV45 in superior parietal region of the left hemisphere - UC Berkeley
66	LH_SUPERIORTEMPORAL_AV45	SUVr for AV45 in superior temporal region of the left hemisphere - UC Berkeley
67	LH_SUPRAMARGINAL_AV45	SUVr for AV45 in supramarginal region of the left hemisphere - UC Berkeley

68	LH_TEMPORALPOLE_AV45	SUVr for AV45 in temporal pole region of the left hemisphere - UC Berkeley
69	LH_TRANSVERSETEMPORAL_AV45	SUVr for AV45 in transverse temporal region of the left hemisphere - UC Berkeley
70	RH_CAUDALANTERIOR CINGULATE_ AV45	SUVr for AV45 in caudal anterior cingulate region of the right hemisphere - UC Berkeley
71	RH_CAUDALMIDDLEFRONTAL_AV45	SUVr for AV45 in caudal middle frontal region of the right hemisphere - UC Berkeley
72	RH_CUNEUS_AV45	SUVr for AV45 in cuneus region of the right hemisphere - UC Berkeley
73	RH_ENTORHINAL_AV45	SUVr for AV45 in entorhinal region of the right hemisphere - UC Berkeley
74	RH_FRONTALPOLE_AV45	SUVr for AV45 in frontal pole region of the right hemisphere - UC Berkeley
75	RH_FUSIFORM_AV45	SUVr for AV45 in frontal pole region of the right hemisphere - UC Berkeley
76	RH_INFERIORPARIETAL_AV45	SUVr for AV45 in inferior parietal region of the right hemisphere - UC Berkeley
77	RH_INFERIORTEMPORAL_AV45	SUVr for AV45 in inferior temporal region of the right hemisphere - UC Berkeley
78	RH_INSULA_AV45	SUVr for AV45 in insula region of the right hemisphere - UC Berkeley
79	RH_ISTHMUSCINGULATE_AV45	SUVr for AV45 in isthmus cingulate region of the right hemisphere - UC Berkeley
80	RH_LATERALOCCIPITAL_AV45	SUVr for AV45 in lateral occipital region of the right hemisphere - UC Berkeley
81	RH_LATERALORBITOFRONTAL_AV45	SUVr for AV45 in lateral orbitofrontal region of the right hemisphere - UC Berkeley
82	RH_LINGUAL_AV45	SUVr for AV45 in lingual region of the right hemisphere - UC Berkeley

83	RH_MEDIALORBITOFRONTAL_AV45	SUVr for AV45 in medial orbitofrontal region of the right hemisphere - UC Berkeley
84	RH_MIDDLETEMPORAL_AV45	SUVr for AV45 in middle temporal region of the right hemisphere - UC Berkeley
85	RH_PARACENTRAL_AV45	SUVr for AV45 in paracentral region of the right hemisphere - UC Berkeley
86	RH_PARAHIPPOCAMPAL_AV45	SUVr for AV45 in parahippocampal region of the right hemisphere - UC Berkeley
87	RH_PARSOPERCULARIS_AV45	SUVr for AV45 in pars opercularis region of the right hemisphere - UC Berkeley
88	RH_PARSORBITALIS_AV45	SUVr for AV45 in pars orbitalis region of the right hemisphere - UC Berkeley
89	RH_PARSTRIANGULARIS_AV45	SUVr for AV45 in pars triangularis region of the right hemisphere - UC Berkeley
90	RH_POSTCENTRAL_AV45	SUVr for AV45 in postcentral region of the right hemisphere - UC Berkeley
91	RH_POSTERIORCINGULATE_AV45	SUVr for AV45 in posterior cingulate region of the right hemisphere - UC Berkeley
92	RH_PRECENTRAL_AV45	SUVr for AV45 in precentral region of the right hemisphere - UC Berkeley
93	RH_PRECUNEUS_AV45	SUVr for AV45 in precuneus region of the right hemisphere - UC Berkeley
94	RH_ROSTRALANTERIOR CINGULATE_AV45	SUVr for AV45 in rostral anterior cingulate region of the right hemisphere - UC Berkeley
95	RH_ROSTRALMIDDLEFRONTAL_AV45	SUVr for AV45 in rostral middle frontal region of the right hemisphere - UC Berkeley
96	RH_SUPERIORFRONTAL_AV45	SUVr for AV45 in superior frontal region of the right hemisphere - UC Berkeley
97	RH_SUPERIORPARIETAL_AV45	SUVr for AV45 in superior parietal region of the right hemisphere - UC Berkeley

98	RH_SUPERIORTEMPORAL_AV45	SUVr for AV45 in superior temporal region of the right hemisphere - UC Berkeley
99	RH_SUPRAMARGINAL_AV45	SUVr for AV45 in supramarginal region of the right hemisphere - UC Berkeley
100	RH_TEMPORALPOLE_AV45	SUVr for AV45 in temporal pole region of the right hemisphere - UC Berkeley
101	RH_TRANSVERSETEMPORAL_AV45	SUVr for AV45 in transverse temporal region of the right hemisphere - UC Berkeley
102	ANGULL01_FDG	Globally normalized CMRgl from Angular L - BAINMRC
103	ANGULR01_FDG	Globally normalized CMRgl from Angular R - BAINMRC
104	ANGULR02_FDG	Globally normalized CMRgl from Angular R - BAINMRC
105	ANGULL02_FDG	Globally normalized CMRgl from Angular L - BAINMRC
106	ANGULR03_FDG	Globally normalized CMRgl from Angular R - BAINMRC
107	ANGULR04_FDG	Globally normalized CMRgl from Angular R - BAINMRC
108	ANGULR05_FDG	Globally normalized CMRgl from Angular R - BAINMRC
109	CINGPSTL01_FDG	Globally normalized CMRgl from Cingulum Post L - BAINMRC
110	CINGPSTL02_FDG	Globally normalized CMRgl from Cingulum Post L - BAINMRC
111	CINGPST03_FDG	Globally normalized CMRgl from Cingulum Post - BAINMRC
112	CINGPST04_FDG	Globally normalized CMRgl from Cingulum Post - BAINMRC

113	CINGPST05_FDG	Globally normalized CMRgl from Cingulum Post - BAINMRC
114	CINGPST07_FDG	Globally normalized CMRgl from Cingulum Post - BAINMRC
115	CINGPST09_FDG	Globally normalized CMRgl from Cingulum Post - BAINMRC
116	CINGPSTR12_FDG	Globally normalized CMRgl from Cingulum Post R - BAINMRC
117	TMPINFR01_FDG	Globally normalized CMRgl from Temporal Inf R - BAINMRC
118	TMPINFL02_FDG	Globally normalized CMRgl from Temporal Inf L - BAINMRC
119	TMPINFL03_FDG	Globally normalized CMRgl from Temporal Inf L - BAINMRC
120	TMPINFR03_FDG	Globally normalized CMRgl from Temporal Inf R - BAINMRC
121	TMPINFL04_FDG	Globally normalized CMRgl from Temporal Inf L - BAINMRC
122	TMPINFR04_FDG	Globally normalized CMRgl from Temporal Inf R - BAINMRC
123	TMPINFR04_FDG	Globally normalized CMRgl from Temporal Inf R - BAINMRC
124	TMPINFL06_FDG	Globally normalized CMRgl from Temporal Inf L - BAINMRC
125	TMPINFR06_FDG	Globally normalized CMRgl from Temporal Inf L - BAINMRC
126	TMPINFL09_FDG	Globally normalized CMRgl from Temporal Inf L - BAINMRC
127	TMPINFL10_FDG	Globally normalized CMRgl from Temporal Inf L - BAINMRC

128	TMPINFL11_FDG	Globally normalized CMRgl from Temporal Inf L - BAINMRC
129	HCI_FDG	Hypometabolic Convergence Index FDG - BAINMRC
130	HCI_2014_FDG	Hypometabolic Convergence Index FDG - BAINMRC

UC Berkeley: University of California Berkeley, BAINMRC: Banner Alzheimer's Institute NMRC Summaries
