# Supplementary material for: Multiscale networks in Alzheimer’s disease identify brain hypometabolism as central across biological scales
Source: PLoS Comput Biol. 2025 Oct 17;21(10):e1013583. doi: 10.1371/journal.pcbi.1013583 (PMC12548887; doi:10.1371/journal.pcbi.1013583)
Supplement: S4 Table — (PDF) [file pcbi.1013583.s004.pdf]

Description of the variables in the MRI dataset

Num	Node	Description
131	CEREB_TCV	Total Cerebrum Cranial Volume (supratentorial portion) (cm^3) - WMH
132	CEREB_TCB	Total Cerebrum Brain Volume (cm^3) - WMH
133	CEREB_TCC	Total Cerebrum Cerebrospinal Fluid Volume (cm^3) - WMH
134	CEREB_GRAY	Total Cerebrum Gray Matter Volume (cm^3) - WMH
135	CEREB_WHITE	Total Cerebrum White Matter Volume (cm^3) - WMH
136	L_HIPPO	Segmented Left Hippocampus Volume (cm^3) - WMH
137	R_HIPPO	Segmented Right Hippocampus Volume (cm^3) - WMH
138	TBM_1	Measure 1 - Numerical summary of cumulative temporal lobe atrophy average within a statistically defined region-of-interest ($p < 0.00001$) inside the temporal lobes; summaries are scaled by 1000 (e.g. 1000: no change, 1200: 20% increase, 800: 20% loss) - TBM
139	TBM_2	Measure 2 - Numerical summary of cumulative temporal lobe atrophy average within a statistically defined region-of-interest ($p < 0.00001$) inside the temporal lobes; summaries are scaled by 1000 (e.g. 1000: no change, 1200: 20% increase, 800: 20% loss) - TBM
140	IPCA	Percent annualized whole brain atrophy detected by iterative principal component Analysis (IPCA) - BAI MRI NMRC
141	LHIPPO	Left Hippocampus Volume (mm^3) Semi-automated hippocampal volumetry using fluid transformation brain Warping software by Surgical Navigation Technologies (SNT) - UCSF
142	RHIPPO	Right Hippocampus Volume (mm^3) Semi-automated hippocampal volumetry using fluid transformation brain Warping software by Surgical Navigation Technologies (SNT) - UCSF
143	ST103CV	Cortical Volume of Right Parahippocampal (mm^3) Cross-Sectional FreeSurfer software - UCSF
144	ST111CV	Cortical Volume of Right Precuneus (mm^3) Cross-Sectional FreeSurfer software - UCSF
145	ST115CV	Cortical Volume of Right Superior Frontal (mm^3) Cross-Sectional FreeSurfer software - UCSF

146	ST116CV	Cortical Volume of Right Superior Parietal (mm^3) Cross-Sectional FreeSurfer software - UCSF
147	ST118CV	Cortical Volume of Right Supramarginal (mm^3) Cross-Sectional FreeSurfer software - UCSF
148	ST119CV	Cortical Volume of Right Temporal Pole (mm^3 Cross-Sectional FreeSurfer software) - UCSF
149	ST12SV	Subcortical Volume of Left Amygdala (mm^3) Cross-Sectional FreeSurfer software - UCSF
150	ST24CV	Cortical Volume of Left Entorhinal (mm^3) Cross-Sectional FreeSurfer software - UCSF
151	ST26CV	Cortical Volume of Left Fusiform (mm^3) Cross-Sectional FreeSurfer software - UCSF
152	ST29SV	Subcortical Volume of Left Hippocampus (mm^3) Cross-Sectional FreeSurfer software - UCSF
153	ST31CV	Cortical Volume of Left Inferior Parietal (mm^3) Cross-Sectional FreeSurfer software - UCSF
154	ST32CV	Cortical Volume of Left Inferior Temporal (mm^3) Cross-Sectional FreeSurfer software - UCSF
155	ST35CV	Cortical Volume of Left Lateral Occipital (mm^3) Cross-Sectional FreeSurfer software - UCSF
156	ST40CV	Cortical Volume of Left Middle Temporal (mm^3) Cross-Sectional FreeSurfer software - UCSF
157	ST44CV	Cortical Volume of Left Parahippocampal (mm^3) Cross-Sectional FreeSurfer software - UCSF
158	ST52CV	Cortical Volume of Left Precuneus (mm^3) Cross-Sectional FreeSurfer software - UCSF
159	ST55CV	Cortical Volume of Left Rostral Middle Frontal (mm^3) Cross-Sectional FreeSurfer software - UCSF
160	ST56CV	Cortical Volume of Left Superior Frontal (mm^3) Cross-Sectional FreeSurfer software - UCSF

161	ST57CV	Cortical Volume of Left Superior Parietal (mm^3) Cross-Sectional FreeSurfer software - UCSF
162	ST59CV	Cortical Volume of Left Supramarginal (mm^3) Cross-Sectional FreeSurfer software - UCSF
163	ST60CV	Cortical Volume of Left Temporal Pole (mm^3) Cross-Sectional FreeSurfer software - UCSF
164	ST71SV	Subcortical Volume of Right Amygdala (mm^3) Cross-Sectional FreeSurfer software - UCSF
165	ST74CV	Cortical Volume of Right Caudal Middle Frontal (mm^3) Cross-Sectional FreeSurfer software - UCSF
166	ST83CV	Cortical Volume of Right Entorhinal (mm^3) Cross-Sectional FreeSurfer software - UCSF
167	ST85CV	Cortical Volume of Right Fusiform (mm^3) Cross-Sectional FreeSurfer software - UCSF
168	ST88SV	Subcortical Volume of Right Hippocampus (mm^3) Cross-Sectional FreeSurfer software - UCSF
169	ST90CV	Cortical Volume of Right Inferior Parietal (mm^3) Cross-Sectional FreeSurfer software - UCSF
170	ST91CV	Cortical Volume of Right Inferior Temporal (mm^3) Cross-Sectional FreeSurfer software - UCSF
171	ST94CV	Cortical Volume of Right Lateral Occipital (mm^3) Cross-Sectional FreeSurfer software - UCSF
172	ST99CV	Cortical Volume of Right Middle Temporal (mm^3) Cross-Sectional FreeSurfer software - UCSF

WMH: University of California Davis - White Matter Hyperintensity Volumes, TBM: University of Southern California - Cross-sectional and longitudinal tensor-based morphometry, BAI MRI NMRC: Banner Alzheimer's Institute MRI NMRC (non-nuclear magnetic resonance cryoporometry), UCSF: University of California San Francisco
