# Supplementary material for: Multiscale networks in Alzheimer’s disease identify brain hypometabolism as central across biological scales
Source: PLoS Comput Biol. 2025 Oct 17;21(10):e1013583. doi: 10.1371/journal.pcbi.1013583 (PMC12548887; doi:10.1371/journal.pcbi.1013583)
Supplement: S5 Table — (PDF) [file pcbi.1013583.s005.pdf]

Description of the variables in the Phenotype dataset

Num	Node	Description
233	MMSE	Mini Mental State Examination, paper-based test with a maximum score of 30, with lower scores indicating more severe cognitive problems
234	MOCA	Montreal Cognitive Assessment test
235	CDR	Clinical Dementia Rate (Normal: 0, Very Slight Dementia: 0.5, Slight Dementia: 1, Moderate Dementia: 2, Severe Dementia: 3)
236	ADSP_DX	Clinical Diagnosis - ADSP
237	ADSP_MEM	Harmonized Composite Memory Score - ADSP
238	ADSP_EXF	Harmonized Composite Executive Function Score - ADSP
239	ADSP_LAN	Harmonized Composite Language Score - ADSP
240	ADSP_VSP	Harmonized Composite Visuospatial Score - ADSP
241	ADAS11	Alzheimer's Disease Assessment Scale Total score 11 (11 task version)
242	ADAS13	Alzheimer's Disease Assessment Scale Total score 13 (which includes all ADAS11 items as well as a test of delayed word recall and a number cancellation or maze task FROM 0-85)
243	UW_MEM	Composite Memory Score - UW
244	UW_EF	Composite Executive Functioning Score - UW

ADSP: Alzheimer's Disease Sequencing Project, UW: University of Washington- Crane Lab
