# Supplementary material for: Multiscale networks in Alzheimer’s disease identify brain hypometabolism as central across biological scales
Source: PLoS Comput Biol. 2025 Oct 17;21(10):e1013583. doi: 10.1371/journal.pcbi.1013583 (PMC12548887; doi:10.1371/journal.pcbi.1013583)
Supplement: S6 Table — (PDF) [file pcbi.1013583.s006.pdf]

Description of the variables in the Risk Factors dataset

Num	Node	Description
173	AXNAUSEA	Nausea - ADSX
174	AXVOMIT	Vomiting - ADSX
175	AXDIARRH	Diarrhea - ADSX
176	AXCONSTP	Constipation - ADSX
177	AXABDOMN	Abdominal Pain - ADSX
178	AXSWEATN	Sweating - ADSX
179	AXDIZZY	Dizziness - ADSX
180	AXENERGY	Low energy - ADSX
181	AXDROWSY	Drowsiness - ADSX
182	AXVISION	Blurred vision - ADSX
183	AXHDACHE	Headache - ADSX
184	AXDRYMTH	Dry Mouth - ADSX
185	AXBREATH	Shortage Of Breath - ADSX
186	AXCOUGH	Coughing - ADSX
187	AXPALPIT	Palpitations - ADSX
188	AXCHEST	Chest pain - ADSX
189	AXURNDIS	Urinary discomfort (e.g., burning) - ADSX
190	AXURNFRQ	Urinary frequency - ADSX
191	AXANKLE	Ankle swelling - ADSX
192	AXMUSCLE	Muscloskeletal Pain - ADSX
193	AXRASH	Rash - ADSX
194	AXINSOMN	Insomnia - ADSX
195	AXDPMOOD	Depressive Mood - ADSX
196	AXCRYING	Crying - ADSX
197	AXELMOOD	Elevated Mood - ADSX
198	AXWANDER	Wandering - ADSX
199	AXFALL	Fall - ADSX
200	HMONSET	Abrupt Onset Dementia - Modified Hachinski
201	HMSTEPWS	Stepwise Deterioration Of Dementia - Modified Hachinski

202	HMSOMATC	Somatic Complaints - Modified Hachinski
203	HMEMOTIO	Emotional Incontinence - Modified Hachinski
204	HMHYPERT	Hypertension - Modified Hachinski
205	HMSTROKE	Stroke - Modified Hachinski
206	HMNEURSM	Focal Neurological Symptoms - Modified Hachinski
207	HMNEURSG	Focal Neurological Signs - Modified Hachinski
208	HMSCORE	Total score - Modified Hachinski
209	PTGENDER	Gender
210	PTDOBYY	Birth year
211	PTHAND	Handedness
212	PTMARRY	Marital status
213	PTEDUCAT	Education
214	GDS	Geriatric Depression Score
215	MHPSYCH	Psychiatric history
216	MH2NEURL	Neurologic History
217	MH3HEAD	Head Eyes Ears Nose Throught History
218	MH4CARD	Cardiovascular history
219	MH5RESP	Respiratory history
220	MH6HEPAT	Hepatic history
221	MH7DERM	Dermatologic, Connective tissue History
222	MH8MUSCL	Musculosketeletal history
223	MH9ENDO	Endocrine history
224	MH10GAST	Gastrointestinal history
225	MH11HEMA	Hematopoietic history
226	MH12RENA	Renal history
227	MH13ALLE	Allergies history
228	MH14ALCH	Alcohol abuse history
229	MH15DRUG	Drug abuse history
230	MH16SMOK	Smoking history
231	MH17MALI	Malignancy history
232	MH18SURG	Major Surgical Procedures history

<u>ADSX</u>: Alzheimer Diagnosis and Symptoms Checklist
