# Supplementary material for: Multiscale networks in Alzheimer’s disease identify brain hypometabolism as central across biological scales
Source: PLoS Comput Biol. 2025 Oct 17;21(10):e1013583. doi: 10.1371/journal.pcbi.1013583 (PMC12548887; doi:10.1371/journal.pcbi.1013583)
Supplement: S7 Table — (PDF) [file pcbi.1013583.s008.pdf]

Detailed connectivity of FDG PET nodes with other network layers

This table lists all significant connections between FDG PET nodes and nodes from other layers in the network. Each row corresponds to a pair of nodes, with columns indicating the FDG PET node, the connected node from another layer, the corresponding layer, and the mutual information (MI) value of the connection.

FDG node	Node from other layer	Layer	Mutual Information
ANGULR01_FDG	EUR_AB42	Molecular	1.0
CINGPSTR12_FDG	ST35CV	MRI	1.0
CINGPST05_FDG	ST56CV	MRI	1.0
CINGPST07_FDG	ST31CV	MRI	1.0
CINGPST07_FDG	ST56CV	MRI	1.0
CINGPST07_FDG	ST59CV	MRI	1.0
CINGPST09_FDG	LHIPPO	MRI	1.0
ANGULL01_FDG	RHIPPO	MRI	1.0
CINGPST09_FDG	ST55CV	MRI	1.0
CINGPSTR12_FDG	IPCA	MRI	1.0
CINGPSTR12_FDG	ST71SV	MRI	1.0
TMPINFR03_FDG	ST119CV	MRI	1.0
TMPINFR01_FDG	UPKelec_PTAU	Molecular	1.0
ANGULR02_FDG	UPKelec_PTAU	Molecular	1.0
ANGULL01_FDG	UPKelec_PTAU	Molecular	1.0
TMPINFR01_FDG	UPKelec_TAU	Molecular	1.0
ANGULR02_FDG	CEREB_TCC	MRI	1.0
TMPINFR01_FDG	IPCA	MRI	1.0
TMPINFL03_FDG	ST90CV	MRI	1.0
TMPINFL03_FDG	ADSP_EXF	Phenotype	1.0
CINGPST05_FDG	R_HIPPO	MRI	1.0
CINGPST05_FDG	CEREB_WHITE	MRI	1.0
CINGPST05_FDG	CEREB_TCB	MRI	1.0

CINGPST04_FDG	CEREB_GRAY	MRI	1.0
ANGULL02_FDG	RHIPPO	MRI	1.0
ANGULR03_FDG	ST24CV	MRI	1.0
ANGULR03_FDG	ST35CV	MRI	1.0
ANGULR03_FDG	ST94CV	MRI	1.0
ANGULR04_FDG	CEREB_TCC	MRI	1.0
ANGULR04_FDG	RHIPPO	MRI	1.0
ANGULR04_FDG	ST57CV	MRI	1.0
ANGULR01_FDG	RHIPPO	MRI	1.0
ANGULR01_FDG	IPCA	MRI	1.0
ANGULR05_FDG	CEREB_TCB	MRI	1.0
ANGULR05_FDG	CEREB_WHITE	MRI	1.0
ANGULR05_FDG	LHIPPO	MRI	1.0
CINGPSTL02_FDG	CEREB_TCB	MRI	1.0
CINGPSTL02_FDG	R_HIPPO	MRI	1.0
CINGPSTL02_FDG	IPCA	MRI	1.0
CINGPSTL02_FDG	LHIPPO	MRI	1.0
CINGPSTL02_FDG	RHIPPO	MRI	1.0
TMPINFR03_FDG	RHIPPO	MRI	1.0
ANGULR04_FDG	UPKelec_AB42	Molecular	1.0
TMPINFR03_FDG	ST52CV	MRI	1.0
TMPINFL11_FDG	IPCA	MRI	1.0
TMPINFR03_FDG	ST71SV	MRI	1.0
ANGULR05_FDG	APP	Molecular	1.0
ANGULL02_FDG	APP	Molecular	1.0
ANGULR02_FDG	APP	Molecular	1.0
TMPINFL10_FDG	ST55CV	MRI	1.0
TMPINFR04_FDG	TL	Molecular	1.0
TMPINFL02_FDG	TL	Molecular	1.0
ANGULL01_FDG	TL	Molecular	1.0
TMPINFL11_FDG	ST40CV	MRI	1.0

TMPINFL10_FDG	IPCA	MRI	1.0
HCI_2014_FDG	CEREB_TCB	MRI	1.0
TMPINFR05_FDG	UGOT_PLASMAPTAU	Molecular	1.0
HCI_2014_FDG	CEREB_TCC	MRI	1.0
HCI_2014_FDG	CEREB_GRAY	MRI	1.0
HCI_2014_FDG	IPCA	MRI	1.0
ANGULL01_FDG	FUJI_AB42	Molecular	1.0
TMPINFR06_FDG	EUR_AB42/40	Molecular	1.0
CINGPSTL01_FDG	EUR_AB42/40	Molecular	1.0
TMPINFL10_FDG	LHIPPO	MRI	1.0
ANGULR02_FDG	IPCA	MRI	1.0
TMPINFL06_FDG	L_HIPPO	MRI	1.0
TMPINFR04_FDG	CEREB_GRAY	MRI	1.0
TMPINFR03_FDG	ST94CV	MRI	1.0
TMPINFL09_FDG	ST55CV	MRI	1.0
TMPINFR03_FDG	ADSP_EXF	Phenotype	1.0
TMPINFL10_FDG	L_HIPPO	MRI	1.0
TMPINFR05_FDG	ST57CV	MRI	1.0
TMPINFR05_FDG	CEREB_TCV	MRI	1.0
TMPINFL06_FDG	LHIPPO	MRI	1.0
TMPINFR04_FDG	IPCA	MRI	1.0
TMPINFL04_FDG	LHIPPO	MRI	1.0
ANGULR04_FDG	ST40CV	MRI	0.999
TMPINFR05_FDG	ST59CV	MRI	0.999
TMPINFR01_FDG	TBM_1	MRI	0.999
HCI_2014_FDG	LHIPPO	MRI	0.999
HCI_2014_FDG	RHIPPO	MRI	0.999
TMPINFL04_FDG	TBM_2	MRI	0.999
ANGULR05_FDG	TBM_1	MRI	0.999
TMPINFL10_FDG	TBM_1	MRI	0.999
TMPINFL10_FDG	ST91CV	MRI	0.999

TMPINFL06_FDG	TBM_1	MRI	0.999
CINGPST09_FDG	ST74CV	MRI	0.999
TMPINFL10_FDG	ST29SV	MRI	0.999
CINGPST04_FDG	IPCA	MRI	0.999
CINGPST04_FDG	LHIPPO	MRI	0.999
CINGPST04_FDG	RHIPPO	MRI	0.999
CINGPST09_FDG	ST40CV	MRI	0.999
TMPINFR06_FDG	ST56CV	MRI	0.999
CINGPST09_FDG	ST29SV	MRI	0.999
CINGPST09_FDG	ST115CV	MRI	0.999
CINGPST05_FDG	ST40CV	MRI	0.999
CINGPST05_FDG	ST91CV	MRI	0.999
HCI_FDG	EUR_AB42	Molecular	0.999
CINGPST05_FDG	TBM_1	MRI	0.999
HCI_2014_FDG	UPKelec_PTAU	Molecular	0.998
CINGPST04_FDG	ST90CV	MRI	0.998
CINGPST04_FDG	ST26CV	MRI	0.998
CINGPST04_FDG	ST111CV	MRI	0.998
CINGPST04_FDG	TBM_2	MRI	0.998
CINGPST04_FDG	TBM_1	MRI	0.998
TMPINFL04_FDG	ST90CV	MRI	0.998
CINGPST03_FDG	TBM_1	MRI	0.998
TMPINFL06_FDG	ST85CV	MRI	0.998
TMPINFR06_FDG	TBM_2	MRI	0.998
ANGULR02_FDG	ST115CV	MRI	0.998
CINGPSTL02_FDG	ST55CV	MRI	0.998
ANGULR02_FDG	ST55CV	MRI	0.998
ANGULR02_FDG	ST99CV	MRI	0.998
CINGPSTL02_FDG	ST115CV	MRI	0.998
CINGPSTL01_FDG	TBM_2	MRI	0.998
ANGULR01_FDG	TBM_1	MRI	0.998

ANGULR01_FDG	TBM_2	MRI	0.998
ANGULR03_FDG	ST91CV	MRI	0.998
CINGPST04_FDG	ST74CV	MRI	0.998
ANGULR02_FDG	TBM_1	MRI	0.998
CINGPST04_FDG	UW_MEM	Phenotype	0.998
CINGPST05_FDG	ST115CV	MRI	0.998
CINGPST05_FDG	ST118CV	MRI	0.998
TMPINFR01_FDG	TBM_2	MRI	0.998
CINGPST05_FDG	ST35CV	MRI	0.998
ANGULR01_FDG	ST55CV	MRI	0.998
TMPINFR01_FDG	ST115CV	MRI	0.998
TMPINFR01_FDG	ST55CV	MRI	0.998
CINGPST04_FDG	ST99CV	MRI	0.998
CINGPST04_FDG	ST91CV	MRI	0.997
TMPINFL09_FDG	ADSP_MEM	Phenotype	0.997
TMPINFR06_FDG	ST85CV	MRI	0.997
TMPINFR06_FDG	ST90CV	MRI	0.997
CINGPSTL02_FDG	L_HIPPO	MRI	0.997
ANGULR05_FDG	ST29SV	MRI	0.997
TMPINFL10_FDG	ST35CV	MRI	0.997
TMPINFL10_FDG	UW_MEM	Phenotype	0.997
TMPINFR01_FDG	ST26CV	MRI	0.997
ANGULR05_FDG	ST31CV	MRI	0.997
ANGULR01_FDG	ST115CV	MRI	0.997
CINGPST09_FDG	UW_MEM	Phenotype	0.997
CINGPSTL02_FDG	ST35CV	MRI	0.997
CINGPSTL02_FDG	ST26CV	MRI	0.997
CINGPSTL02_FDG	ST31CV	MRI	0.997
CINGPSTL02_FDG	ST40CV	MRI	0.997
TMPINFR01_FDG	ST56CV	MRI	0.997
TMPINFR01_FDG	ST85CV	MRI	0.997

CINGPSTL02_FDG	ST56CV	MRI	0.997
CINGPSTL02_FDG	ST85CV	MRI	0.997
TMPINFR01_FDG	ST99CV	MRI	0.997
CINGPSTL02_FDG	ST99CV	MRI	0.997
TMPINFL02_FDG	ST40CV	MRI	0.997
TMPINFL02_FDG	ST56CV	MRI	0.997
ANGULL02_FDG	ST31CV	MRI	0.997
TMPINFR05_FDG	ADSP_EXF	Phenotype	0.997
CINGPST05_FDG	ST29SV	MRI	0.997
ANGULL02_FDG	ST40CV	MRI	0.997
ANGULR01_FDG	ST31CV	MRI	0.997
ANGULR02_FDG	TBM_2	MRI	0.997
ANGULR01_FDG	ST56CV	MRI	0.997
ANGULR01_FDG	ST57CV	MRI	0.997
ANGULR02_FDG	ST85CV	MRI	0.997
TMPINFR06_FDG	ST52CV	MRI	0.996
TMPINFL03_FDG	ST59CV	MRI	0.996
CINGPST04_FDG	ADSP_EXF	Phenotype	0.996
CINGPST04_FDG	ADSP_MEM	Phenotype	0.996
CINGPST04_FDG	ST29SV	MRI	0.996
ANGULR01_FDG	ST26CV	MRI	0.996
CINGPSTL02_FDG	ST90CV	MRI	0.996
ANGULR02_FDG	ST111CV	MRI	0.996
ANGULR01_FDG	ST99CV	MRI	0.996
CINGPSTL02_FDG	ST74CV	MRI	0.996
CINGPSTL02_FDG	ST32CV	MRI	0.996
CINGPSTL02_FDG	ST118CV	MRI	0.996
CINGPSTL01_FDG	ST31CV	MRI	0.996
TMPINFL03_FDG	ST116CV	MRI	0.996
ANGULL01_FDG	ST56CV	MRI	0.996
CINGPST05_FDG	ST88SV	MRI	0.996

ANGULR05_FDG	ST88SV	MRI	0.996
TMPINFL02_FDG	ST32CV	MRI	0.996
TMPINFL02_FDG	ST26CV	MRI	0.996
CINGPST07_FDG	TBM_1	MRI	0.996
ANGULL01_FDG	ST40CV	MRI	0.996
ANGULL01_FDG	ST32CV	MRI	0.996
CINGPST07_FDG	UW_EF	Phenotype	0.996
TMPINFR01_FDG	ST94CV	MRI	0.996
TMPINFR01_FDG	ST90CV	MRI	0.996
ANGULR01_FDG	ST74CV	MRI	0.996
ANGULL01_FDG	ST31CV	MRI	0.996
ANGULL01_FDG	ST26CV	MRI	0.996
TMPINFR01_FDG	ST111CV	MRI	0.996
ANGULR01_FDG	ST40CV	MRI	0.996
CINGPSTL01_FDG	ST26CV	MRI	0.996
CINGPSTL02_FDG	ST111CV	MRI	0.996
ANGULR01_FDG	ST118CV	MRI	0.996
ANGULL02_FDG	ST111CV	MRI	0.995
CINGPSTL01_FDG	ST111CV	MRI	0.995
ANGULL01_FDG	ST111CV	MRI	0.995
ANGULR02_FDG	ST91CV	MRI	0.995
ANGULR02_FDG	ST90CV	MRI	0.995
CINGPSTL02_FDG	ST59CV	MRI	0.995
ANGULR01_FDG	ST111CV	MRI	0.995
CINGPSTL02_FDG	ST91CV	MRI	0.995
TMPINFR01_FDG	ST91CV	MRI	0.995
ANGULR01_FDG	ST116CV	MRI	0.995
TMPINFL06_FDG	ST29SV	MRI	0.995
TMPINFR01_FDG	ST116CV	MRI	0.995
ANGULR01_FDG	ST90CV	MRI	0.995
ANGULR01_FDG	ST91CV	MRI	0.995

ANGULL01_FDG	ST90CV	MRI	0.995
CINGPSTR12_FDG	UPplasma_AB42	Molecular	0.994
CINGPSTL02_FDG	UW_MEM	Phenotype	0.994
ANGULL02_FDG	UW_MEM	Phenotype	0.994
TMPINFR04_FDG	ADSP_EXF	Phenotype	0.994
TMPINFR04_FDG	ST88SV	MRI	0.994
CINGPSTL02_FDG	ST52CV	MRI	0.994
ANGULL01_FDG	ST59CV	MRI	0.994
CINGPSTR12_FDG	ST83CV	MRI	0.994
ANGULL01_FDG	UW_MEM	Phenotype	0.994
TMPINFL02_FDG	ST59CV	MRI	0.994
CINGPSTR12_FDG	ADSP_LAN	Phenotype	0.994
ANGULL02_FDG	ST52CV	MRI	0.994
ANGULR01_FDG	ST59CV	MRI	0.994
CINGPSTL01_FDG	UW_MEM	Phenotype	0.994
ANGULR01_FDG	ST52CV	MRI	0.994
TMPINFL11_FDG	ST12SV	MRI	0.994
CINGPST04_FDG	ST52CV	MRI	0.993
CINGPST04_FDG	ST88SV	MRI	0.993
ANGULR02_FDG	ADSP_MEM	Phenotype	0.993
ANGULR05_FDG	ST111CV	MRI	0.993
ANGULL01_FDG	ST52CV	MRI	0.993
CINGPST05_FDG	ST111CV	MRI	0.993
TMPINFL06_FDG	ADSP_EXF	Phenotype	0.993
TMPINFR01_FDG	ADSP_MEM	Phenotype	0.992
CINGPSTL02_FDG	ADSP_MEM	Phenotype	0.992
TMPINFL03_FDG	UPK_TAU	Molecular	0.992
HCI_2014_FDG	ST115CV	MRI	0.992
TMPINFR03_FDG	ADSP_LAN	Phenotype	0.992
HCI_2014_FDG	ST56CV	MRI	0.992
TMPINFR06_FDG	ADSP_MEM	Phenotype	0.992

TMPINFR06_FDG	ADSP_EXF	Phenotype	0.992
HCI_FDG	APP	Molecular	0.991
ANGULL02_FDG	ADSP_MEM	Phenotype	0.991
CINGPSTL02_FDG	ADSP_EXF	Phenotype	0.991
ANGULL02_FDG	ADSP_EXF	Phenotype	0.991
ANGULL01_FDG	ADSP_MEM	Phenotype	0.991
TMPINFR01_FDG	ADSP_EXF	Phenotype	0.991
HCI_2014_FDG	ST55CV	MRI	0.991
HCI_2014_FDG	ST90CV	MRI	0.991
HCI_2014_FDG	ST31CV	MRI	0.991
CINGPSTL01_FDG	ADSP_MEM	Phenotype	0.991
ANGULR02_FDG	ADSP_EXF	Phenotype	0.991
TMPINFR05_FDG	UPplasma_AB42	Molecular	0.991
ANGULR01_FDG	ADSP_MEM	Phenotype	0.991
HCI_2014_FDG	ST35CV	MRI	0.99
ANGULR05_FDG	ST44CV	MRI	0.99
HCI_2014_FDG	ST32CV	MRI	0.99
CINGPST04_FDG	ST83CV	MRI	0.99
CINGPSTL02_FDG	ST29SV	MRI	0.99
TMPINFR01_FDG	ST88SV	MRI	0.99
CINGPSTL01_FDG	ADSP_EXF	Phenotype	0.99
CINGPSTL02_FDG	ST88SV	MRI	0.99
ANGULR01_FDG	ADSP_EXF	Phenotype	0.99
HCI_2014_FDG	ST91CV	MRI	0.99
ANGULL01_FDG	ADSP_EXF	Phenotype	0.99
ANGULR01_FDG	ST88SV	MRI	0.989
ANGULR02_FDG	ST24CV	MRI	0.989
TMPINFR04_FDG	ST119CV	MRI	0.989
HCI_2014_FDG	ST57CV	MRI	0.989
ANGULL02_FDG	ST29SV	MRI	0.989
ANGULR01_FDG	ST29SV	MRI	0.989

HCI_2014_FDG	ST116CV	MRI	0.989
HCI_2014_FDG	ST99CV	MRI	0.989
HCI_2014_FDG	ST40CV	MRI	0.989
CINGPST07_FDG	ST52CV	MRI	0.989
HCI_2014_FDG	ST94CV	MRI	0.988
HCI_2014_FDG	ST85CV	MRI	0.988
HCI_2014_FDG	R_HIPPO	MRI	0.988
ANGULR01_FDG	ST24CV	MRI	0.988
ANGULR02_FDG	ST88SV	MRI	0.988
HCI_2014_FDG	ST111CV	MRI	0.988
CINGPSTL02_FDG	ST24CV	MRI	0.988
HCI_2014_FDG	L_HIPPO	MRI	0.988
TMPINFR01_FDG	ST24CV	MRI	0.988
HCI_2014_FDG	ST118CV	MRI	0.987
HCI_2014_FDG	TBM_1	MRI	0.987
CINGPSTL01_FDG	ST60CV	MRI	0.987
HCI_2014_FDG	ST26CV	MRI	0.987
CINGPSTL01_FDG	ST24CV	MRI	0.987
HCI_2014_FDG	ST59CV	MRI	0.987
CINGPST05_FDG	ST12SV	MRI	0.986
CINGPSTL02_FDG	ST83CV	MRI	0.986
HCI_2014_FDG	ST52CV	MRI	0.986
TMPINFR01_FDG	ST83CV	MRI	0.986
HCI_2014_FDG	UW_MEM	Phenotype	0.985
ANGULL01_FDG	ST83CV	MRI	0.985
ANGULR02_FDG	ST119CV	MRI	0.985
TMPINFL04_FDG	TS_RATIO	Molecular	0.985
ANGULR01_FDG	ST83CV	MRI	0.985
CINGPSTL01_FDG	ST83CV	MRI	0.985
TMPINFR01_FDG	ST103CV	MRI	0.984
CINGPSTL02_FDG	ST103CV	MRI	0.984

HCI_2014_FDG	ST74CV	MRI	0.984
ANGULL02_FDG	FUJI_AB42/40	Molecular	0.983
ANGULR01_FDG	ST119CV	MRI	0.983
TMPINFR01_FDG	ST44CV	MRI	0.983
TMPINFL02_FDG	ST44CV	MRI	0.983
TMPINFL04_FDG	UPK_TAU	Molecular	0.983
CINGPSTL02_FDG	ST44CV	MRI	0.983
ANGULR01_FDG	ST103CV	MRI	0.983
ANGULR04_FDG	UPK_TAU	Molecular	0.983
HCI_2014_FDG	FUJI_AB42	Molecular	0.982
HCI_FDG	CEREB_GRAY	MRI	0.982
HCI_FDG	CEREB_WHITE	MRI	0.981
HCI_2014_FDG	ADSP_MEM	Phenotype	0.981
ANGULR02_FDG	ST12SV	MRI	0.98
ANGULR02_FDG	UPK_TAU	Molecular	0.98
ANGULR01_FDG	ST71SV	MRI	0.978
ANGULL02_FDG	ST71SV	MRI	0.978
TMPINFR03_FDG	UPplasma_AB42	Molecular	0.978
CINGPSTL02_FDG	ST71SV	MRI	0.978
TMPINFL02_FDG	ST71SV	MRI	0.978
ANGULL01_FDG	UPK_TAU	Molecular	0.978
TMPINFR01_FDG	UPK_TAU	Molecular	0.978
CINGPST05_FDG	UPK_PTAU	Molecular	0.977
TMPINFL02_FDG	UPK_TAU	Molecular	0.977
ANGULR01_FDG	UPK_TAU	Molecular	0.977
HCI_FDG	FUJI_AB42	Molecular	0.976
CINGPSTL02_FDG	ST12SV	MRI	0.975
HCI_2014_FDG	ST29SV	MRI	0.975
ANGULL02_FDG	ST12SV	MRI	0.975
CINGPST04_FDG	UPK_PTAU	Molecular	0.974
ANGULR02_FDG	UPK_PTAU	Molecular	0.974

ANGULR01_FDG	ST12SV	MRI	0.974
HCI_2014_FDG	TBM_2	MRI	0.974
ANGULR04_FDG	UPK_PTAU	Molecular	0.974
HCI_2014_FDG	ST88SV	MRI	0.974
CINGPST04_FDG	BACE	Molecular	0.973
TMPINFR06_FDG	UPK_PTAU	Molecular	0.97
ANGULR02_FDG	UW_EF	Phenotype	0.968
TMPINFL09_FDG	UPK_AB42	Molecular	0.968
TMPINFR01_FDG	UPK_PTAU	Molecular	0.967
ANGULL01_FDG	UPK_PTAU	Molecular	0.967
ANGULR01_FDG	UPK_PTAU	Molecular	0.966
HCI_2014_FDG	ST24CV	MRI	0.965
HCI_FDG	ST31CV	MRI	0.965
HCI_FDG	ST91CV	MRI	0.964
CINGPST04_FDG	ADSP_LAN	Phenotype	0.964
HCI_FDG	ST32CV	MRI	0.964
HCI_2014_FDG	ST60CV	MRI	0.964
TMPINFR04_FDG	TS_RATIO	Molecular	0.963
HCI_FDG	ST40CV	MRI	0.963
HCI_FDG	ST59CV	MRI	0.962
HCI_2014_FDG	ST83CV	MRI	0.962
CINGPSTL02_FDG	UW_EF	Phenotype	0.962
TMPINFR01_FDG	UW_EF	Phenotype	0.962
ANGULL01_FDG	UW_EF	Phenotype	0.961
HCI_FDG	ST52CV	MRI	0.961
ANGULR01_FDG	UW_EF	Phenotype	0.961
HCI_FDG	ST26CV	MRI	0.961
HCI_2014_FDG	ST119CV	MRI	0.96
TMPINFR04_FDG	ADSP_LAN	Phenotype	0.96
TMPINFL03_FDG	ADAS11	Phenotype	0.959
HCI_FDG	UW_MEM	Phenotype	0.959

CINGPSTL02_FDG	UPplasma_AB42	Molecular	0.958
ANGULL02_FDG	UPplasma_AB42	Molecular	0.958
HCI_2014_FDG	ST44CV	MRI	0.957
TMPINFL03_FDG	ADAS13	Phenotype	0.957
HCI_2014_FDG	ADSP_EXF	Phenotype	0.957
HCI_FDG	ADSP_MEM	Phenotype	0.956
HCI_2014_FDG	ST103CV	MRI	0.955
TMPINFR04_FDG	UPK_AB42	Molecular	0.952
TMPINFR06_FDG	ADSP_LAN	Phenotype	0.95
HCI_FDG	ST29SV	MRI	0.95
HCI_FDG	ST88SV	MRI	0.948
ANGULL01_FDG	BACE	Molecular	0.948
HCI_2014_FDG	ST71SV	MRI	0.946
TMPINFR01_FDG	ADSP_LAN	Phenotype	0.945
ANGULL01_FDG	ADSP_LAN	Phenotype	0.945
CINGPSTL02_FDG	ADSP_LAN	Phenotype	0.945
ANGULR01_FDG	ADSP_LAN	Phenotype	0.945
CINGPSTL01_FDG	ADSP_LAN	Phenotype	0.944
HCI_2014_FDG	ST12SV	MRI	0.944
HCI_2014_FDG	UPK_TAU	Molecular	0.941
HCI_FDG	ST24CV	MRI	0.94
HCI_FDG	ST60CV	MRI	0.939
TMPINFR04_FDG	TS_RATIO_ADJ	Molecular	0.938
HCI_FDG	ST83CV	MRI	0.937
HCI_FDG	ST119CV	MRI	0.935
HCI_2014_FDG	UPK_PTAU	Molecular	0.933
HCI_FDG	ADSP_EXF	Phenotype	0.933
TMPINFL09_FDG	ADAS13	Phenotype	0.931
HCI_2014_FDG	UW_EF	Phenotype	0.93
ANGULL02_FDG	UPK_AB42	Molecular	0.926
ANGULR01_FDG	UPK_AB42	Molecular	0.926

ANGULL01_FDG	UPK_AB42	Molecular	0.926
HCI_FDG	ST12SV	MRI	0.919
HCI_FDG	ST71SV	MRI	0.919
HCI_2014_FDG	ADSP_LAN	Phenotype	0.919
ANGULR04_FDG	ADAS13	Phenotype	0.917
CINGPST04_FDG	ADAS13	Phenotype	0.916
ANGULL01_FDG	TS_RATIO_ADJ	Molecular	0.909
CINGPST04_FDG	ADAS11	Phenotype	0.906
HCI_FDG	UW_EF	Phenotype	0.904
TMPINFR04_FDG	ADAS13	Phenotype	0.9
TMPINFL06_FDG	ADAS13	Phenotype	0.899
HCI_FDG	ADSP_LAN	Phenotype	0.893
TMPINFL10_FDG	ADAS11	Phenotype	0.886
ANGULR02_FDG	ADAS13	Phenotype	0.883
TMPINFR01_FDG	ADAS13	Phenotype	0.869
ANGULL02_FDG	ADAS13	Phenotype	0.869
ANGULR01_FDG	ADAS13	Phenotype	0.869
CINGPSTL02_FDG	ADAS13	Phenotype	0.869
TMPINFL02_FDG	ADAS13	Phenotype	0.869
TMPINFR04_FDG	ADAS11	Phenotype	0.868
ANGULL01_FDG	ADAS13	Phenotype	0.868
TMPINFL06_FDG	ADAS11	Phenotype	0.867
ANGULR02_FDG	ADAS11	Phenotype	0.857
TMPINFR03_FDG	ADSP_VSP	Phenotype	0.854
HCI_2014_FDG	UPK_AB42	Molecular	0.852
HCI_2014_FDG	ADAS13	Phenotype	0.851
TMPINFL02_FDG	ADAS11	Phenotype	0.84
TMPINFR01_FDG	ADAS11	Phenotype	0.84
CINGPSTL02_FDG	ADAS11	Phenotype	0.84
CINGPSTL01_FDG	ADAS11	Phenotype	0.839
ANGULL01_FDG	ADAS11	Phenotype	0.839

ANGULR01_FDG	ADAS11	Phenotype	0.839
HCI_FDG	UPK_AB42	Molecular	0.831
ANGULR03_FDG	PTDOBYY	Risk Factors	0.829
HCI_2014_FDG	FUJI_AB42/40	Molecular	0.826
HCI_FDG	FUJI_AB42/40	Molecular	0.824
HCI_2014_FDG	ADAS11	Phenotype	0.819
TMPINFL03_FDG	MMSE	Phenotype	0.775
TMPINFR03_FDG	MMSE	Phenotype	0.775
ANGULR02_FDG	PTDOBYY	Risk Factors	0.721
TMPINFL02_FDG	PTDOBYY	Risk Factors	0.705
ANGULL02_FDG	PTDOBYY	Risk Factors	0.705
CINGPSTL02_FDG	PTDOBYY	Risk Factors	0.705
TMPINFR05_FDG	ADSP_VSP	Phenotype	0.682
TMPINFL09_FDG	MMSE	Phenotype	0.673
TMPINFL10_FDG	MMSE	Phenotype	0.673
CINGPST05_FDG	MMSE	Phenotype	0.668
CINGPST04_FDG	ADSP_VSP	Phenotype	0.666
CINGPST04_FDG	MMSE	Phenotype	0.653
HCI_2014_FDG	PTDOBYY	Risk Factors	0.648
HCI_2014_FDG	MOCA	Phenotype	0.64
ANGULR02_FDG	ADSP_VSP	Phenotype	0.629
TMPINFR04_FDG	MMSE	Phenotype	0.624
ANGULR02_FDG	MMSE	Phenotype	0.618
TMPINFR01_FDG	MMSE	Phenotype	0.606
CINGPSTL02_FDG	MMSE	Phenotype	0.606
ANGULR01_FDG	MMSE	Phenotype	0.605
ANGULL01_FDG	MMSE	Phenotype	0.604
TMPINFR01_FDG	ADSP_VSP	Phenotype	0.601
ANGULR01_FDG	ADSP_VSP	Phenotype	0.6
TMPINFR01_FDG	PTEDUCAT	Risk Factors	0.531
TMPINFR05_FDG	GDS	Risk Factors	0.509

HCI_2014_FDG	ADSP_VSP	Phenotype	0.498
HCI_2014_FDG	MMSE	Phenotype	0.495
CINGPST09_FDG	GDS	Risk Factors	0.488
CINGPST05_FDG	GDS	Risk Factors	0.466
HCI_FDG	MMSE	Phenotype	0.459
CINGPST04_FDG	CDR	Phenotype	0.405
ANGULR02_FDG	CDR	Phenotype	0.371
TMPINFR01_FDG	CDR	Phenotype	0.352
ANGULR01_FDG	CDR	Phenotype	0.352
CINGPSTL02_FDG	CDR	Phenotype	0.352
ANGULL02_FDG	CDR	Phenotype	0.351
ANGULL01_FDG	CDR	Phenotype	0.351
TMPINFR06_FDG	CDR	Phenotype	0.345
TMPINFL10_FDG	CDR	Phenotype	0.323
ANGULR02_FDG	ADSP_DX	Phenotype	0.319
HCI_FDG	GDS	Risk Factors	0.317
TMPINFL04_FDG	HMSCORE	Risk Factors	0.311
ANGULR04_FDG	HMSCORE	Risk Factors	0.311
ANGULR01_FDG	ADSP_DX	Phenotype	0.307
TMPINFR01_FDG	ADSP_DX	Phenotype	0.306
ANGULL01_FDG	ADSP_DX	Phenotype	0.306
CINGPSTL02_FDG	ADSP_DX	Phenotype	0.306
ANGULL02_FDG	ADSP_DX	Phenotype	0.305
CINGPSTL01_FDG	ADSP_DX	Phenotype	0.305
CINGPST07_FDG	MH3HEAD	Risk Factors	0.302
CINGPST04_FDG	ADSP_DX	Phenotype	0.286
HCI_2014_FDG	CDR	Phenotype	0.285
TMPINFL11_FDG	PTMARRY	Risk Factors	0.284
CINGPST07_FDG	AXMUSCLE	Risk Factors	0.273
TMPINFL11_FDG	PTGENDER	Risk Factors	0.267
TMPINFR05_FDG	ADSP_DX	Phenotype	0.262

TMPINFR01_FDG	HMSCORE	Risk Factors	0.261
CINGPST03_FDG	MH18SURG	Risk Factors	0.261
ANGULR01_FDG	HMSCORE	Risk Factors	0.26
HCI_2014_FDG	ADSP_DX	Phenotype	0.259
CINGPST07_FDG	AXENERGY	Risk Factors	0.259
ANGULR03_FDG	MH13ALLE	Risk Factors	0.258
HCI_FDG	CDR	Phenotype	0.256
TMPINFL09_FDG	HMHYPERT	Risk Factors	0.256
ANGULR03_FDG	MH8MUSCL	Risk Factors	0.255
TMPINFL10_FDG	MH12RENA	Risk Factors	0.254
ANGULR03_FDG	AXENERGY	Risk Factors	0.248
CINGPSTR12_FDG	AXDPMOOD	Risk Factors	0.247
CINGPST05_FDG	ADSP_DX	Phenotype	0.247
CINGPST07_FDG	AXDPMOOD	Risk Factors	0.241
ANGULR04_FDG	HMHYPERT	Risk Factors	0.238
TMPINFL04_FDG	HMHYPERT	Risk Factors	0.238
CINGPST09_FDG	MH16SMOK	Risk Factors	0.237
CINGPST04_FDG	HMHYPERT	Risk Factors	0.236
TMPINFR05_FDG	MH7DERM	Risk Factors	0.233
CINGPST09_FDG	PTGENDER	Risk Factors	0.23
TMPINFL10_FDG	MH3HEAD	Risk Factors	0.23
CINGPST07_FDG	AXURNFRQ	Risk Factors	0.23
TMPINFL10_FDG	PTGENDER	Risk Factors	0.23
HCI_2014_FDG	HMSCORE	Risk Factors	0.228
ANGULR04_FDG	MH8MUSCL	Risk Factors	0.224
HCI_FDG	ADSP_DX	Phenotype	0.223
CINGPST09_FDG	AXMUSCLE	Risk Factors	0.222
CINGPST07_FDG	AXDROWSY	Risk Factors	0.218
CINGPST05_FDG	AXMUSCLE	Risk Factors	0.217
CINGPST03_FDG	AXURNFRQ	Risk Factors	0.215
ANGULR03_FDG	AXURNFRQ	Risk Factors	0.215

TMPINFL06_FDG	MH16SMOK	Risk Factors	0.214
TMPINFR04_FDG	MH8MUSCL	Risk Factors	0.212
ANGULR05_FDG	MH2NEURL	Risk Factors	0.208
TMPINFR01_FDG	HMHYPERT	Risk Factors	0.208
CINGPSTL02_FDG	MH12RENA	Risk Factors	0.208
CINGPST07_FDG	MH5RESP	Risk Factors	0.205
TMPINFL06_FDG	PTGENDER	Risk Factors	0.205
HCI_2014_FDG	PTMARRY	Risk Factors	0.204
CINGPST07_FDG	PTMARRY	Risk Factors	0.202
TMPINFL09_FDG	MH4CARD	Risk Factors	0.202
TMPINFL09_FDG	AXURNFRQ	Risk Factors	0.202
TMPINFL04_FDG	MH4CARD	Risk Factors	0.201
TMPINFL04_FDG	MH17MALI	Risk Factors	0.201
ANGULR04_FDG	MH4CARD	Risk Factors	0.201
TMPINFL06_FDG	MH2NEURL	Risk Factors	0.2
ANGULR02_FDG	MH3HEAD	Risk Factors	0.2
ANGULR01_FDG	MH9ENDO	Risk Factors	0.199
TMPINFR04_FDG	MH18SURG	Risk Factors	0.197
TMPINFR01_FDG	PTGENDER	Risk Factors	0.197
CINGPSTL02_FDG	MH3HEAD	Risk Factors	0.197
CINGPSTL02_FDG	PTGENDER	Risk Factors	0.197
CINGPSTR12_FDG	PTMARRY	Risk Factors	0.196
TMPINFL02_FDG	MH8MUSCL	Risk Factors	0.195
CINGPSTL02_FDG	MHPSYCH	Risk Factors	0.191
TMPINFR04_FDG	MH17MALI	Risk Factors	0.188
TMPINFR06_FDG	MH18SURG	Risk Factors	0.184
CINGPST04_FDG	AXURNFRQ	Risk Factors	0.178
TMPINFL06_FDG	AXURNFRQ	Risk Factors	0.178
HCI_2014_FDG	MH10GAST	Risk Factors	0.173
HCI_2014_FDG	PTGENDER	Risk Factors	0.173
ANGULL01_FDG	MH18SURG	Risk Factors	0.172

ANGULR03_FDG	AXCONSTP	Risk Factors	0.171
CINGPSTL02_FDG	MH5RESP	Risk Factors	0.162
CINGPSTL01_FDG	AXENERGY	Risk Factors	0.161
HCI_2014_FDG	MH8MUSCL	Risk Factors	0.16
TMPINFR04_FDG	AXENERGY	Risk Factors	0.153
HCI_2014_FDG	MH18SURG	Risk Factors	0.144
TMPINFR05_FDG	MH11HEMA	Risk Factors	0.139
HCI_FDG	MH10GAST	Risk Factors	0.138
HCI_FDG	MH8MUSCL	Risk Factors	0.135
CINGPST09_FDG	AXABDOMN	Risk Factors	0.135
CINGPST07_FDG	AXSWEATN	Risk Factors	0.135
TMPINFR01_FDG	AXDPMOOD	Risk Factors	0.133
CINGPSTL01_FDG	AXDPMOOD	Risk Factors	0.133
TMPINFR05_FDG	AXABDOMN	Risk Factors	0.131
HCI_FDG	MHPSYCH	Risk Factors	0.129
ANGULR01_FDG	AXDROWSY	Risk Factors	0.126
CINGPSTL01_FDG	AXDROWSY	Risk Factors	0.126
ANGULL01_FDG	AXDROWSY	Risk Factors	0.126
TMPINFL11_FDG	AXCOUGH	Risk Factors	0.122
TMPINFL11_FDG	AXFALL	Risk Factors	0.122
TMPINFL11_FDG	AXDIZZY	Risk Factors	0.122
TMPINFR03_FDG	AXDIARRH	Risk Factors	0.118
ANGULR02_FDG	AXDIZZY	Risk Factors	0.11
CINGPSTL02_FDG	AXCOUGH	Risk Factors	0.108
TMPINFR01_FDG	AXCOUGH	Risk Factors	0.108
CINGPST09_FDG	AXCONSTP	Risk Factors	0.103
CINGPST09_FDG	AXANKLE	Risk Factors	0.103
CINGPSTL02_FDG	AXINSOMN	Risk Factors	0.102
ANGULL01_FDG	AXCONSTP	Risk Factors	0.099
CINGPST05_FDG	AXANKLE	Risk Factors	0.091
TMPINFR03_FDG	AXWANDER	Risk Factors	0.089

TMPINFR06_FDG	PTHAND	Risk Factors	0.088
HCI_FDG	AXCONSTP	Risk Factors	0.086
ANGULR01_FDG	MH11HEMA	Risk Factors	0.085
CINGPST07_FDG	AXABDOMN	Risk Factors	0.084
ANGULL01_FDG	MH11HEMA	Risk Factors	0.083
TMPINFR05_FDG	AXVISION	Risk Factors	0.082
HCI_2014_FDG	PTHAND	Risk Factors	0.08
ANGULL02_FDG	PTHAND	Risk Factors	0.08
CINGPSTL01_FDG	AXRASH	Risk Factors	0.076
TMPINFL11_FDG	AXHDACHE	Risk Factors	0.075
CINGPST09_FDG	AXFALL	Risk Factors	0.074
TMPINFL10_FDG	AXSWEATN	Risk Factors	0.074
TMPINFL09_FDG	AXHDACHE	Risk Factors	0.074
CINGPST03_FDG	AXELMOOD	Risk Factors	0.072
CINGPSTR12_FDG	AXBREATH	Risk Factors	0.07
TMPINFR01_FDG	AXCRYING	Risk Factors	0.068
CINGPSTL01_FDG	AXCRYING	Risk Factors	0.068
TMPINFL06_FDG	AXANKLE	Risk Factors	0.063
ANGULR05_FDG	AXSWEATN	Risk Factors	0.063
CINGPST03_FDG	AXABDOMN	Risk Factors	0.053
CINGPST07_FDG	AXBREATH	Risk Factors	0.05
HCI_2014_FDG	MH6HEPAT	Risk Factors	0.045
TMPINFR06_FDG	HMSOMATC	Risk Factors	0.039
ANGULR02_FDG	HMSOMATC	Risk Factors	0.039
CINGPST05_FDG	HMNEURSG	Risk Factors	0.037
TMPINFR01_FDG	AXELMOOD	Risk Factors	0.037
CINGPSTL02_FDG	AXURNDIS	Risk Factors	0.037
ANGULR02_FDG	MH14ALCH	Risk Factors	0.034
CINGPST03_FDG	MH15DRUG	Risk Factors	0.031
TMPINFR01_FDG	AXVOMIT	Risk Factors	0.029
TMPINFR01_FDG	AXWANDER	Risk Factors	0.029

CINGPST05_FDG	AXVOMIT	Risk Factors	0.027
TMPINFR04_FDG	AXWANDER	Risk Factors	0.027
HCI_2014_FDG	AXWANDER	Risk Factors	0.023
TMPINFL09_FDG	HMSTEPWS	Risk Factors	0.021
ANGULR04_FDG	HMNEURSG	Risk Factors	0.014
ANGULR04_FDG	HMSTEPWS	Risk Factors	0.014
TMPINFR01_FDG	HMNEURSM	Risk Factors	0.011
TMPINFR06_FDG	HMNEURSM	Risk Factors	0.01
ANGULR02_FDG	MH15DRUG	Risk Factors	0.008
ANGULL02_FDG	MH15DRUG	Risk Factors	0.006
HCI_2014_FDG	HMONSET	Risk Factors	0.005
