# Supplementary material for: Multiscale networks in Alzheimer’s disease identify brain hypometabolism as central across biological scales
Source: PLoS Comput Biol. 2025 Oct 17;21(10):e1013583. doi: 10.1371/journal.pcbi.1013583 (PMC12548887; doi:10.1371/journal.pcbi.1013583)
Supplement: S8 Table — (PDF) [file pcbi.1013583.s009.pdf]

Top 10 genetic input paths in the control group.

Out of the 30,000 total paths identified from participants in the cohort, 6,787 did not appear at all in 100 realizations of the simulations in the permuted paths. The top paths (those that passed the test for negative controls) for genetic input are shown in the following table.

Path	*sum_count*
APOE_A2 → APOE → PHS → CDR	209
APOE_A2 → APOE → PHS → ADSP_VSP	208
APOE_A1 → APOE_A2 → APOE → MMSE	175
APOE_A1 → APOE_A2 → APOE → ADSP_MEM	173
APOE_A1 → APOE_A2 → APOE → CDR	169
APOE_A1 → APOE_A2 → APOE → ADAS13	168
APOE_A1 → APOE_A2 → APOE → ADSP_LAN	168
APOE_A1 → APOE_A2 → APOE → ADSP_EXF	168
APOE_A1 → APOE_A2 → APOE → UW_MEM	168
APOE_A1 → APOE_A2 → APOE → ADAS11	168

This table lists, out of the 388 paths identified in the control group based on genetic layer inputs, the top 10 paths. Paths are ranked by their cross-correlations scores, with the *sum_count* indicating the cumulative frequency of node pair occurrences within each path. The path more commonly found is APOE_A2 - APOE - PHS → CDR.
