# Supplementary material for: Multiscale networks in Alzheimer’s disease identify brain hypometabolism as central across biological scales
Source: PLoS Comput Biol. 2025 Oct 17;21(10):e1013583. doi: 10.1371/journal.pcbi.1013583 (PMC12548887; doi:10.1371/journal.pcbi.1013583)
Supplement: S9 Table — (PDF) [file pcbi.1013583.s010.pdf]

Top 10 molecular input paths in the control group.

Out of the 30,000 total paths identified from participants in the cohort, 6,787 did not appear at all in 100 realizations of the simulations in the permuted paths. The top paths (those that passed the test for negative controls) for molecular input are shown in the following table.

Path	*sum_count*
TS_RATIO → TL → TS_RATIO_ADJ → CDR	190
TS_RATIO → TL → TS_RATIO_ADJ → MMSE	189
TS_RATIO → TL → TS_RATIO_ADJ → ADSP_MEM	189
TS_RATIO → TL → TS_RATIO_ADJ → ADSP_EXF	189
TS_RATIO → TL → TS_RATIO_ADJ → UW_MEM	189
TS_RATIO → TS_RATIO_ADJ → TL → CDR	134
TS_RATIO_ADJ → TL → ADSP_EXF → ADSP_MEM	119
TS_RATIO_ADJ → TL → ADSP_EXF → MOCA	119
TS_RATIO_ADJ → TL → ADSP_EXF → UW_MEM	119
TS_RATIO_ADJ → TL → ADSP_EXF → ADSP_LAN	119

This table lists, out of the 1232 paths identified in the control group based on molecular layer inputs, the top 10 paths. Paths are ranked by their cross-correlations scores, with the *sum_count* indicating the cumulative frequency of node pair occurrences within each path. The path more commonly found is TS_RATIO - TL - TS_RATIO_ADJ → CDR.
