# Supplementary material for: Multiscale networks in Alzheimer’s disease identify brain hypometabolism as central across biological scales
Source: PLoS Comput Biol. 2025 Oct 17;21(10):e1013583. doi: 10.1371/journal.pcbi.1013583 (PMC12548887; doi:10.1371/journal.pcbi.1013583)
Supplement: S10 Table — (PDF) [file pcbi.1013583.s011.pdf]

Top 10 PET input paths in the control group.

Out of the 30,000 total paths identified from participants in the cohort, 6,787 did not appear at all in 100 realizations of the simulations in the permuted paths. The top paths (those that passed the test for negative controls) for PET input are shown in the following table.

Path	*sum_count*
RH_ENTORHINAL_AV45 → ANGULR04_FDG → UPK_TAU → ADAS11	202
RH_ENTORHINAL_AV45 → ANGULR04_FDG → UPK_TAU → MOCA	202
RH_ENTORHINAL_AV45 → ANGULR04_FDG → UPK_TAU → MMSE	202
RH_ENTORHINAL_AV45 → ANGULR04_FDG → UPK_TAU → ADSP_VSP	202
RH_ENTORHINAL_AV45 → ANGULR04_FDG → UPK_TAU → ADAS13	202
RH_ENTORHINAL_AV45 → ANGULR04_FDG → UPK_TAU → ADSP_EXF	202
RH_ENTORHINAL_AV45 → ANGULR04_FDG → UPK_TAU → UW_EF	202
RH_ENTORHINAL_AV45 → ANGULR04_FDG → UPK_TAU → UW_MEM	202
RH_ENTORHINAL_AV45 → ANGULR04_FDG → UPK_TAU → CDR	202
RH_ENTORHINAL_AV45 → ANGULR04_FDG → UPK_TAU → ADSP_MEM	202

This table lists, out of the 2799 paths identified in the control group based on PET layer inputs, the top 10 paths. Paths are ranked by their cross-correlations scores, with the *sum_count* indicating the cumulative frequency of node pair occurrences within each path. The path more commonly found is RH_ENTORHINAL_AV45 - ANGULR04_FDG → UPK_TAU → any node of the phenotype layer.
