# Supplementary material for: Multiscale networks in Alzheimer’s disease identify brain hypometabolism as central across biological scales
Source: PLoS Comput Biol. 2025 Oct 17;21(10):e1013583. doi: 10.1371/journal.pcbi.1013583 (PMC12548887; doi:10.1371/journal.pcbi.1013583)
Supplement: S11 Table — (PDF) [file pcbi.1013583.s012.pdf]

Top 10 MRI input paths in the control group.

Out of the 30,000 total paths identified from participants in the cohort, 6,787 did not appear at all in 100 realizations of the simulations in the permuted paths. The top paths (those that passed the test for negative controls) for MRI input are shown in the following table.

Path	*sum_count*
TBM_2 → TBM_1 → COMPOSITE_AV45 → MOCA	120
TBM_2 → TBM_1 → COMPOSITE_AV45 → ADSP_LAN	120
TBM_2 → TBM_1 → COMPOSITE_AV45 → ADSP_EXF	120
TBM_2 → TBM_1 → COMPOSITE_AV45 → ADAS11	120
TBM_2 → TBM_1 → COMPOSITE_AV45 → ADSP_MEM	120
TBM_2 → TBM_1 → COMPOSITE_AV45 → ADAS13	120
TBM_2 → TBM_1 → COMPOSITE_AV45 → ADSP_VSP	120
TBM_2 → TBM_1 → COMPOSITE_AV45 → MMSE	120
TBM_2 → TBM_1 → COMPOSITE_AV45 → UW_EF	120
TBM_2 → TBM_1 → LH_POSTCENTRAL_AV45 → ADAS13	119

This table lists, out of the 468 paths identified in the control group based on MRI layer inputs, the top 10 paths. Paths are ranked by their cross-correlations scores, with the *sum_count* indicating the cumulative frequency of node pair occurrences within each path. The path more commonly found is TBM_2 - TBM_1 → COMPOSITE_AV45 → any node of the phenotype layer.
