# Supplementary material for: Multiscale networks in Alzheimer’s disease identify brain hypometabolism as central across biological scales
Source: PLoS Comput Biol. 2025 Oct 17;21(10):e1013583. doi: 10.1371/journal.pcbi.1013583 (PMC12548887; doi:10.1371/journal.pcbi.1013583)
Supplement: S12 Table — (PDF) [file pcbi.1013583.s013.pdf]

Top 10 risk factors input paths in the control group.

Out of the 30,000 total paths identified from participants in the cohort, 6,787 did not appear at all in 100 realizations of the simulations in the permuted paths. The top paths (those that passed the test for negative controls) for risk factors input are shown in the following table.

Path	*sum_count*
AXBREATH → AXANKLE → AXPALPIT → MOCA	182
AXBREATH → AXANKLE → AXPALPIT → ADAS13	181
AXBREATH → AXANKLE → AXPALPIT → ADAS11	180
AXBREATH → AXANKLE → AXPALPIT → UW_EF	180
AXBREATH → AXANKLE → AXPALPIT → ADSP_MEM	179
AXBREATH → AXANKLE → AXPALPIT → ADSP_EXF	179
AXPALPIT → AXANKLE → AXBREATH → ADAS13	136
AXPALPIT → AXANKLE → AXBREATH → ADAS11	136
AXPALPIT → AXANKLE → AXBREATH → MMSE	136
AXPALPIT → AXANKLE → AXBREATH → UW_MEM	136

This table lists, out of the 1900 paths identified in the control group based on risk factors layer inputs, the top 10 paths. Paths are ranked by their cross-correlations scores, with the *sum_count* indicating the cumulative frequency of node pair occurrences within each path. The path more commonly found is AXBREATH - AXANKLE - AXPALPIT → MOCA.
