# Supplementary material for: Multiscale networks in Alzheimer’s disease identify brain hypometabolism as central across biological scales
Source: PLoS Comput Biol. 2025 Oct 17;21(10):e1013583. doi: 10.1371/journal.pcbi.1013583 (PMC12548887; doi:10.1371/journal.pcbi.1013583)
Supplement: S13 Table — (PDF) [file pcbi.1013583.s014.pdf]

Top 10 genetic input paths in the MCI group.

Out of the 30,000 total paths identified from participants in the cohort, 7,304 did not appear at all in 100 realizations of the simulations in the permuted paths. The top paths (those that passed the test for negative controls) for genetic input are shown in the following table.

Path	*sum_count*
TOMM40_A1 → TOMM40_A2 → BRAAK34_SUVR → MOCA	116
TOMM40_A1 → TOMM40_A2 → BRAAK34_SUVR → MMSE	116
TOMM40_A1 → TOMM40_A2 → BRAAK34_SUVR → UW_EF	116
TOMM40_A1 → TOMM40_A2 → BRAAK34_SUVR → ADAS13	116
TOMM40_A1 → TOMM40_A2 → BRAAK34_SUVR → ADSP_EXF	116
TOMM40_A1 → TOMM40_A2 → BRAAK34_SUVR → ADSP_MEM	116
TOMM40_A1 → TOMM40_A2 → BRAAK34_SUVR → ADSP_LAN	116
TOMM40_A1 → TOMM40_A2 → BRAAK34_SUVR → UW_MEM	116
TOMM40_A1 → TOMM40_A2 → BRAAK34_SUVR → ADSP_VSP	116
TOMM40_A1 → TOMM40_A2 → BRAAK1_SUVR → ADSP_MEM	115

This table lists, out of the 213 paths identified in the MCI group based on genetic layer inputs, the top 10 paths. Paths are ranked by their cross-correlations scores, with the *sum_count* indicating the cumulative frequency of node pair occurrences within each path. The path more commonly found is TOMM40_A1 - TOMM40_A2 → BRAAK34_SUVR → any node of the phenotype layer.
