# Supplementary material for: Multiscale networks in Alzheimer’s disease identify brain hypometabolism as central across biological scales
Source: PLoS Comput Biol. 2025 Oct 17;21(10):e1013583. doi: 10.1371/journal.pcbi.1013583 (PMC12548887; doi:10.1371/journal.pcbi.1013583)
Supplement: S14 Table — (PDF) [file pcbi.1013583.s015.pdf]

Top 10 molecular input paths in the MCI group.

Out of the 30,000 total paths identified from participants in the cohort, 7,304 did not appear at all in 100 realizations of the simulations in the permuted paths. The top paths (those that passed the test for negative controls) for molecular input are shown in the following table.

Path	*sum_count*
UPK_AB42 → EUR_AB42/40 → ANGULR01_FDG → MMSE	121
UPK_AB42 → EUR_AB42/40 → ANGULR01_FDG → UW_EF	121
UPK_AB42 → EUR_AB42/40 → ANGULR01_FDG → MOCA	121
UPK_AB42 → EUR_AB42/40 → ANGULR01_FDG → ADSP_MEM	121
TL → TMPINFR01_FDG → APP → MMSE	121
TL → TMPINFR01_FDG → APP → MOCA	121
TL → TMPINFR01_FDG → APP → ADSP_MEM	121
TL → TMPINFR01_FDG → APP → ADSP_EXF	121
TL → TMPINFR01_FDG → APP → ADSP_LAN	121
TL → TMPINFR01_FDG → APP → ADAS11	121

This table lists, out of the 1136 paths identified in the MCI group based on molecular layer inputs, the top 10 paths. Paths are ranked by their cross-correlations scores, with the *sum_count* indicating the cumulative frequency of node pair occurrences within each path. The path more commonly found is UPK_AB42 - EUR_AB42/40 → ANGULR01_FDG → MMSE, UW_EF, MOCA and ADSP_MEM.
