# Supplementary material for: Multiscale networks in Alzheimer’s disease identify brain hypometabolism as central across biological scales
Source: PLoS Comput Biol. 2025 Oct 17;21(10):e1013583. doi: 10.1371/journal.pcbi.1013583 (PMC12548887; doi:10.1371/journal.pcbi.1013583)
Supplement: S15 Table — (PDF) [file pcbi.1013583.s016.pdf]

Top 10 PET input paths in the MCI group.

Out of the 30,000 total paths identified from participants in the cohort, 7,304 did not appear at all in 100 realizations of the simulations in the permuted paths. The top paths (those that passed the test for negative controls) for PET input are shown in the following table.

Path	*sum_count*
ANGULR05_FDG → AXCRYING → AXDPMOOD → MOCA	250
ANGULR05_FDG → AXCRYING → AXDPMOOD → MMSE	246
ANGULR05_FDG → AXCRYING → AXDPMOOD → ADSP_VSP	246
ANGULR05_FDG → AXCRYING → AXDPMOOD → UW_EF	244
ANGULR05_FDG → AXCRYING → AXDPMOOD → ADAS11	244
ANGULR05_FDG → AXCRYING → AXDPMOOD → ADAS13	243
ANGULR05_FDG → AXCRYING → AXDPMOOD → UW_MEM	239
ANGULR05_FDG → AXCRYING → AXDPMOOD → ADSP_MEM	239
ANGULR05_FDG → AXCRYING → AXDPMOOD → ADSP_LAN	238
ANGULR05_FDG → AXCRYING → AXDPMOOD → CDR	236

This table lists, out of the 1985 paths identified in the MCI group based on PET layer inputs, the top 10 paths. Paths are ranked by their cross-correlations scores, with the *sum_count* indicating the cumulative frequency of node pair occurrences within each path. The path more commonly found is ANGULR05_FDG → AXCRYING - AXDPMOOD → MOCA.
