# Supplementary material for: Multiscale networks in Alzheimer’s disease identify brain hypometabolism as central across biological scales
Source: PLoS Comput Biol. 2025 Oct 17;21(10):e1013583. doi: 10.1371/journal.pcbi.1013583 (PMC12548887; doi:10.1371/journal.pcbi.1013583)
Supplement: S16 Table — (PDF) [file pcbi.1013583.s017.pdf]

Top 10 MRI input paths in the MCI group.

Out of the 30,000 total paths identified from participants in the cohort, 7,304 did not appear at all in 100 realizations of the simulations in the permuted paths. The top paths (those that passed the test for negative controls) for MRI input are shown in the following table.

Path	*sum_count*
CEREB_TCC → TMPINFL04_FDG → MOCA → MMSE	170
CEREB_TCC → TMPINFL04_FDG → MOCA → ADSP_MEM	169
CEREB_TCC → TMPINFL04_FDG → MOCA → ADSP_LAN	169
CEREB_TCC → TMPINFL04_FDG → MOCA → UW_MEM	169
CEREB_TCC → TMPINFL04_FDG → MOCA → ADSP_VSP	168
CEREB_TCC → TMPINFL04_FDG → MOCA → ADSP_EXF	168
CEREB_TCC → TMPINFL04_FDG → MOCA → CDR	167
CEREB_TCC → TMPINFL04_FDG → MOCA → UW_EF	167
CEREB_TCC → TMPINFL04_FDG → MOCA	163
CEREB_TCC → TMPINFL04_FDG → MH8MUSCL → CDR	155

This table lists, out of the 749 paths identified in the MCI group based on MRI layer inputs, the top 10 paths. Paths are ranked by their cross-correlations scores, with the *sum_count* indicating the cumulative frequency of node pair occurrences within each path. The path more commonly found is CEREB_TCC → TMPINFL04_FDG → MOCA - MMSE.
