# Supplementary material for: Multiscale networks in Alzheimer’s disease identify brain hypometabolism as central across biological scales
Source: PLoS Comput Biol. 2025 Oct 17;21(10):e1013583. doi: 10.1371/journal.pcbi.1013583 (PMC12548887; doi:10.1371/journal.pcbi.1013583)
Supplement: S17 Table — (PDF) [file pcbi.1013583.s018.pdf]

Top 10 risk factors input paths in the MCI group.

Out of the 30,000 total paths identified from participants in the cohort, 7,304 did not appear at all in 100 realizations of the simulations in the permuted paths. The top paths (those that passed the test for negative controls) for risk factors input are shown in the following table.

Path	*sum_count*
HMNEURSG → HMNEURSM → TMPINFL04_FDG → MOCA → ADSP_MEM	309
HMNEURSG → HMNEURSM → TMPINFL04_FDG → MOCA → ADSP_LAN	309
HMNEURSG → HMNEURSM → TMPINFL04_FDG → MOCA → UW_EF	308
HMNEURSG → HMNEURSM → TMPINFL04_FDG → MOCA → MMSE	308
HMNEURSG → HMNEURSM → TMPINFL04_FDG → MOCA → ADSP_EXF	308
HMNEURSG → HMNEURSM → TMPINFL04_FDG → MOCA → UW_MEM	307
HMNEURSG → HMNEURSM → TMPINFL04_FDG → MOCA → ADSP_VSP	306
HMNEURSG → HMNEURSM → TMPINFL04_FDG → MOCA → CDR	302
AXHDACHE → HMNEURSM → TMPINFL04_FDG → MOCA	297
HMNEURSG → HMNEURSM → TMPINFL04_FDG → MOCA	297

Table 1: **Top 10 risk factors input paths in the MCI group**. This table lists, out of the 3221 paths identified in the MCI group based on risk factors layer inputs, the top 10 paths. Paths are ranked by their cross-correlations scores, with the *sum_count* indicating the cumulative frequency of node pair occurrences within each path. The path more commonly found is HMNEURSG - HMNEURSM → TMPINFL04_FDG → MOCA - ADSP_MEM, ADSP_LAN.
