# Supplementary material for: Multiscale networks in Alzheimer’s disease identify brain hypometabolism as central across biological scales
Source: PLoS Comput Biol. 2025 Oct 17;21(10):e1013583. doi: 10.1371/journal.pcbi.1013583 (PMC12548887; doi:10.1371/journal.pcbi.1013583)
Supplement: S18 Table — (PDF) [file pcbi.1013583.s019.pdf]

Top 10 genetic input paths in the AD group.

Out of the 30,000 total paths identified from participants in the cohort, 6,930 did not appear at all in 100 realizations of the simulations in the permuted paths. The top paths (those that passed the test for negative controls) for genetic input are shown in the following table.

Path	*sum_count*
APOE_A1 → CINGPST07_FDG → GDS → UW_EF	114
APOE_A1 → CINGPST07_FDG → GDS → ADAS13	114
APOE_A1 → CINGPST07_FDG → MH11HEMA → ADSP_LAN	114
APOE_A1 → CINGPST07_FDG → MH11HEMA → ADSP_EXF	114
APOE_A1 → CINGPST07_FDG → MH11HEMA → ADSP_MEM	114
APOE_A1 → CINGPST07_FDG → MH11HEMA → MMSE	114
APOE_A1 → CINGPST07_FDG → MH11HEMA → UW_EF	114
APOE_A1 → CINGPST07_FDG → MH11HEMA → MOCA	114
APOE_A1 → CINGPST07_FDG → CINGPST04_FDG → UW_MEM	113
APOE_A1 → CINGPST07_FDG → CINGPST04_FDG → CDR	113

This table lists, out of the 383 paths identified in the AD group based on genetic layer inputs, the top 10 paths. Paths are ranked by their cross-correlations scores, with the *sum_count* indicating the cumulative frequency of node pair occurrences within each path. The path more commonly found is APOE_A1 → CINGPST07_FDG → GDS → UW_EF, ADAS13.
