# Supplementary material for: Multiscale networks in Alzheimer’s disease identify brain hypometabolism as central across biological scales
Source: PLoS Comput Biol. 2025 Oct 17;21(10):e1013583. doi: 10.1371/journal.pcbi.1013583 (PMC12548887; doi:10.1371/journal.pcbi.1013583)
Supplement: S19 Table — (PDF) [file pcbi.1013583.s020.pdf]

Top 10 molecular input paths in the AD group.

Out of the 30,000 total paths identified from participants in the cohort, 6,930 did not appear at all in 100 realizations of the simulations in the permuted paths. The top paths (those that passed the test for negative controls) for molecular input are shown in the following table.

Path	*sum_count*
UPplasma_AB42 → BACE → ANGULL01_FDG → UW_MEM	178
BACE → ANGULL01_FDG → UPKelec_PTAU → ADSP_EXF	126
BACE → ANGULL01_FDG → ANGULL02_FDG → ADSP_LAN	125
BACE → ANGULL01_FDG → ANGULL02_FDG → ADSP_EXF	125
BACE → ANGULL01_FDG → ANGULL02_FDG → MMSE	123
BACE → ANGULL01_FDG → APP → ADAS13	121
BACE → ANGULL01_FDG → APP → MOCA	121
BACE → ANGULL01_FDG → APP → UW_EF	121
BACE → ANGULL01_FDG → APP → ADAS11	121
BACE → ANGULL01_FDG → APP → MMSE	121

This table lists, out of the 1106 paths identified in the AD group based on molecular layer inputs, the top 10 paths. Paths are ranked by their cross-correlations scores, with the *sum_count* indicating the cumulative frequency of node pair occurrences within each path. The path more commonly found is UPplasma_AB42 - BACE → ANGULL01_FDG → UW_MEM.
