# Supplementary material for: Multiscale networks in Alzheimer’s disease identify brain hypometabolism as central across biological scales
Source: PLoS Comput Biol. 2025 Oct 17;21(10):e1013583. doi: 10.1371/journal.pcbi.1013583 (PMC12548887; doi:10.1371/journal.pcbi.1013583)
Supplement: S20 Table — (PDF) [file pcbi.1013583.s021.pdf]

Top 10 PET input paths in the AD group.

Out of the 30,000 total paths identified from participants in the cohort, 6,930 did not appear at all in 100 realizations of the simulations in the permuted paths. The top paths (those that passed the test for negative controls) for PET input are shown in the following table.

Path	*sum_count*
ANGULL02_FDG → ANGULL01_FDG → BACE → ADSP_VSP	215
ANGULL02_FDG → ANGULL01_FDG → BACE → ADSP_LAN	214
ANGULL02_FDG → ANGULL01_FDG → BACE → MOCA	213
ANGULL02_FDG → ANGULL01_FDG → BACE → ADSP_MEM	213
ANGULL02_FDG → ANGULL01_FDG → BACE → ADSP_EXF	209
ANGULL01_FDG → BACE → TMPINFL09_FDG → UW_EF	207
ANGULL02_FDG → ANGULL01_FDG → BACE → MMSE	204
TMPINFR04_FDG → BACE → TMPINFL09_FDG → UW_EF	204
ANGULL02_FDG → ANGULL01_FDG → BACE → UW_MEM	199
BRAAK56_SUVR → UW_MEM → TMPINFL09_FDG → UW_EF	196

This table lists, out of the 3635 paths identified in the AD group based on PET layer inputs, the top 10 paths. Paths are ranked by their cross-correlations scores, with the *sum_count* indicating the cumulative frequency of node pair occurrences within each path. The path more commonly found is ANGULL02_FDG - ANGULL01_FDG → BACE → ADSP_VSP.
