# Supplementary material for: Multiscale networks in Alzheimer’s disease identify brain hypometabolism as central across biological scales
Source: PLoS Comput Biol. 2025 Oct 17;21(10):e1013583. doi: 10.1371/journal.pcbi.1013583 (PMC12548887; doi:10.1371/journal.pcbi.1013583)
Supplement: S21 Table — (PDF) [file pcbi.1013583.s022.pdf]

Top 10 MRI input paths in the AD group.

Out of the 30,000 total paths identified from participants in the cohort, 6,930 did not appear at all in 100 realizations of the simulations in the permuted paths. The top paths (those that passed the test for negative controls) for MRI input are shown in the following table.

Path	*sum_count*
CEREB_TCC → TMPINFR01_FDG → MH4CARD → ADSP_MEM	112
CEREB_TCC → TMPINFR01_FDG → MH4CARD → ADSP_LAN	112
CEREB_TCC → TMPINFR01_FDG → MH4CARD → ADSP_EXF	111
CEREB_TCC → TMPINFR01_FDG → MH4CARD → UW_EF	111
CEREB_TCC → TMPINFR01_FDG → MH4CARD → ADSP_VSP	111
CEREB_TCC → TMPINFR01_FDG → MH4CARD → MOCA	111
CEREB_TCC → TMPINFR01_FDG → MH4CARD → MMSE	111
CEREB_TCC → TMPINFR01_FDG → MH4CARD → ADAS11	110
CEREB_TCC → TMPINFR01_FDG → MH4CARD → CDR	110
IPCA → TMPINFL11_FDG → TL → CDR	109

This table lists, out of the 486 paths identified in the AD group based on MRI layer inputs, the top 10 paths. Paths are ranked by their cross-correlations scores, with the *sum_count* indicating the cumulative frequency of node pair occurrences within each path. The path more commonly found is CEREB_TCC → TMPINFR01_FDG → MH4CARD → ADSP_MEM - ADSP_LAN.
