# Supplementary material for: Multiscale networks in Alzheimer’s disease identify brain hypometabolism as central across biological scales
Source: PLoS Comput Biol. 2025 Oct 17;21(10):e1013583. doi: 10.1371/journal.pcbi.1013583 (PMC12548887; doi:10.1371/journal.pcbi.1013583)
Supplement: S22 Table — (PDF) [file pcbi.1013583.s023.pdf]

Top 10 risk factors input paths in the AD group.

Out of the 30,000 total paths identified from participants in the cohort, 6,930 did not appear at all in 100 realizations of the simulations in the permuted paths. The top paths (those that passed the test for negative controls) for risk factors input are shown in the following table.

Path	*sum_count*
AXELMOOD → MH15DRUG → HMONSET → CDR	152
AXELMOOD → MH15DRUG → HMONSET → ADSP_MEM	152
AXELMOOD → MH15DRUG → HMONSET → UW_EF	152
AXELMOOD → MH15DRUG → HMONSET → ADAS11	152
AXELMOOD → MH15DRUG → HMONSET → ADAS13	152
AXELMOOD → MH15DRUG → HMONSET → MMSE	151
AXELMOOD → MH15DRUG → HMONSET → ADSP_LAN	151
AXELMOOD → MH15DRUG → HMONSET → ADSP_EXF	150
AXELMOOD → MH15DRUG → HMONSET → ADSP_VSP	150
AXELMOOD → MH15DRUG → HMONSET → UW_MEM	148

This table lists, out of the 1320 paths identified in the AD group based on risk factors layer inputs, the top 10 paths. Paths are ranked by their cross-correlations scores, with the *sum_count* indicating the cumulative frequency of node pair occurrences within each path. The path more commonly found is AXELMOOD - MH15DRUG - HMONSET → CDR, ADSP_MEM, UW_EF, ADAS11, ADAS13.
