# Supplementary material for: Multiscale networks in Alzheimer’s disease identify brain hypometabolism as central across biological scales
Source: PLoS Comput Biol. 2025 Oct 17;21(10):e1013583. doi: 10.1371/journal.pcbi.1013583 (PMC12548887; doi:10.1371/journal.pcbi.1013583)
Supplement: Attachment — Submitted filename: Response to Reviewers.pdf [file pcbi.1013583.s025.pdf]

Additional Editor Comments:

Additionally to addressing the reviewer comments, the code for this work does not seem to be available (only the networks). Please provide access to the code according to the PLOS Computational Biology policies (https://journals.plos.org/ploscompbiol/s/code-availability)

To address this, we have ensured that the code relevant to this paper is clearly available and accessible. Specifically, we have:

- Updated the GitHub repository to include all scripts and documentation related to the analyses presented in the paper.
- Added a direct link to the repository containing the code in the manuscript (see page 25, lines 668-670).

Reviewer's Responses to Questions

Reviewer #1:

1. What are the main claims of the paper and how significant are they for the discipline?

The paper suggests that Alzheimer's disease (AD) can be better understood using a systems biology approach that integrates genomic, molecular, and clinical data through multilayer network analysis. This approach highlights key connections between biological features and clinical symptoms, offering a comprehensive framework that could improve diagnostics and personalized treatments.

2. Are these claims novel? If not, which published articles weaken the claims of originality of this one?

While the approach of using systems biology and network analysis is innovative, it is not entirely novel. However, the integration of diverse biological scales (genomics, imaging, risk factors, etc.) could be considered a more novel aspect of this research.

3. Are the claims properly placed in the context of the previous literature? Have the authors treated the literature fairly?

The claims are supported by existing literature, with references to studies on brain hypometabolism and cardiovascular history in AD.

4. Do the data and analyses fully support the claims? If not, what other evidence is required?

The data support the claims, particularly in linking biological pathways to AD features. However, the cross-sectional design and single cohort (ADNI) limit causal conclusions. Longitudinal data and validation in other cohorts could further strengthen the findings.

5. Would additional work improve the paper? How much better would the paper be if this work were performed and how difficult would it be to do this work?

Additional work, such as using longitudinal data, larger sample sizes, and validation in other cohorts, would strengthen the paper. However, obtaining longitudinal data could be challenging due to the need for extended follow-up and more resources.

6. PLOS Computational Biology encourages authors to publish detailed protocols and algorithms as supporting information online. Do any particular methods used in the manuscript warrant such treatment? If a protocol is already provided, for example for a randomized controlled trial, are there any important deviations from it? If so, have the authors explained adequately why the deviations occurred?

The GitHub link to the networks generated is given. However, I had trouble finding the relevant code for this paper.

As mentioned above, the code is now available in page 25, lines 668-670.

7. Are original data deposited in appropriate repositories and accession/version numbers provided for genes, proteins, mutants, diseases, etc.?

The manuscript does not explicitly mention whether the original data have been deposited in public repositories. The GitHub link is given along with the networks generated for the paper. However, I had trouble finding the relevant code for this paper.

The original data from the ADNI project is available at https://adni.loni.usc.edu.As mentioned above, the code is now available in page 25, lines 668-670.

8. Has the author-generated code that underpins the findings been made publicly available?

The GitHub link to the networks generated is given. However, I had trouble finding the relevant code for this paper.

As mentioned above, the code is now available in page 25, lines 668-670.

9. Does the study conform to any relevant guidelines such as CONSORT, MIAME, QUORUM, STROBE, and the Fort Lauderdale agreement?

The manuscript does not explicitly state adherence to CONSORT, MIAME, QUORUM, STROBE, or the Fort Lauderdale agreement. However, it details its methodology, data sources, and ethical considerations. Contacting the authors could confirm this.

The ADNI study (https://adni.loni.usc.edu) is a large, longitudinal, observational cohort study focused on Alzheimer's disease. Based on the ADNI Data Sharing and Publication Policy (https://adni.loni.usc.edu/wp-content/uploads/how_to_apply/ADNI_DSP_Policy.pdf), there is no explicit requirement or statement that ADNI itself adheres to either STROBE or CONSORT reporting guidelines. The policy emphasizes data sharing, citation, and manuscript review, but does not mandate the use of these reporting standards.

10. Are details of the methodology sufficient to allow the experiments to be reproduced?

In my opinion, the manuscript provides methodological details for reproducibility, including data from ADNI, network construction methods, statistical analyses, and

GitHub-hosted code. However, full replication may require additional details on preprocessing, software versions, and parameter settings.

The code is now available in page 25, lines 668-670.

11. Is any software created by the authors freely available?

I had trouble finding the GitHub link. The only links provided in the article include the networks.

The code is now available in page 25, lines 668-670.

12. Is the manuscript well organized and written clearly enough to be accessible to non-specialists?

The manuscript is well-structured and informative.

13. Does the paper use standardized scientific nomenclature and abbreviations? If not, are these explained at the first usage?

Yes, the paper uses standardized scientific nomenclature and abbreviations. Moreover, the supplementary file contains all the abbreviations relevant to the study. However, terms such as "Aβ," "PET," and "MRI" are widely recognized in the field and clarifying these abbreviations upon their first use would improve understanding, especially for readers unfamiliar with the topic.

The abbreviations indicated have been clarified in their first use. Specifically,

- MRI (Magnetic Resonance Imaging), PET (Positron emission tomography) and CSF (cerebrospinal fluid) on page 3, lines 65-66
- fluorodeoxyglucose (FDG) on page 1, in the Abstract

Reviewer #2: In the manuscript, Lara-Simon et al. presents a systems biology study applying multilayer network analysis to integrate multi-layered AD data (genomics, CSF/plasma biomarkers, PET/MRI imaging, clinical phenotypes, and risk factors) to investigate the complex interplay across different biological scales. The authors construct mutual-information-based network for each layer and use the dynamical Boolean simulations to identify potential paths that link molecular changes to clinical phenotype for AD. Via network and path analysis, they conclude that FDG PET hypometabolism may be as a central hub connecting different layers in AD phenotypes (including cognitive impairment and risk factors). This is an interesting study that integrate microscopic and macroscopic layers to address the AD complexity. However, I have some concerns for both methodology and results.

Major issues:

1. The title emphasizes "brain hypometabolism," but this key finding is not mentioned in the Abstract. In addition, in the entire Results section, only lines 305-307 claimed MRI or PET connections associated with metabolic processes. The authors primarily claim hypometabolism findings in the Discussion section. Is there additional analysis that supports this primary conclusion? Given that MRI or PET provided most of the

variables used for network construction (SI sheets), do the network methods justify the variable count across different layers?

In the new version we describe our finding on brain hypometabolism, as a key feature of Alzheimer disease across biological scales, in the abstract, results and discussion (page 1 in the Abstract, page 2 in the Author summary, pages 16, 19, 21 lines 437-439, 471-472, 510-513, 530-532). We have specifically analyzed the role of hypometabolism in the network paths identified in the study.

It is true that some networks (MRI and PET) contains more nodes and for this reason it is more likely paths will involve more nodes in such networks. However, the specificity of the path identification is no influenced by node density at each layer, based on our permutation analysis.

2. Since the multilayer network approach has already been explored in a previous study (reference 11), the authors should state what methodological novelty in this manuscript.

The results presented in this paper can be interpreted as further validation of the method introduced in the reference mentioned by the Reviewer. The novelty of this manuscript lies on the application of that methodology to a substantially different type of dataset, with distinct statistical properties and constraints, and with different clinical implications. A paragraph with one of the differences has been included on page 5, lines 163-167

3. The study aims to address heterogeneity but primarily focuses on paths explaining the phenotype. The manuscript only highlights the network changes in AD, it would better to include MCI and Control as well and compare the differences from network and path perspectives.

We thank the reviewer for this important suggestion. While the original manuscript presented path analyses on the full cohort including all diagnostic groups, we have now extended this analysis to separately compute and compare the top paths within each diagnostic subgroup: control, mild cognitive impairment (MCI), and Alzheimer's disease (AD) patients. For each group, we applied the same network pipeline, Boolean simulation and path scoring methodology across all input layers (genetic, molecular, PET, MRI, and risk factors), allowing a direct comparison of path structures and their prevalence among groups. The top 10 paths for each input and diagnostic group, along with their associated sum_counts (defined as the sum of the counts of each pair of nodes appearing in the path), are provided in the SI (Supplementary Tables 8–22). These subgroup-specific analyses highlight both shared and distinct network mechanisms that characterize disease progression and heterogeneity in AD, and we have added a brief discussion to these results in the revised manuscript (pages 14-15, 21-23, lines 398-406, 502-522, 556-564).

4.In the path analysis, some phenotypes, such as MOCA and 5 risk factors (HMSCORE, AXDIARRH, AXENERGY, AXDPMOOD, AXCRYING) will be linked regardless of the start point is. How to explain this result?

Indeed, our results show that certain variables — notably MOCA and several patient-reported symptoms from the risk factor layer — appear consistently across inferred paths, independent of the input layer. We have clarified and expanded on this point in the revised manuscript, both in the Results and Discussion sections (pages 19, 23, lines 473-483, 568-580), as detailed below:

For these variables, their recurrent presence can be explained by the methodology used for path selection. As described in the Methods section, paths were identified by simulating time-varying Boolean activity across the network and computing temporal cross-correlations between the input signal and all other nodes. The final paths were selected based on a path score, defined as the sum of the inverse of the maximum cross-correlations along each edge. These variables consistently displayed high levels of temporal cross-correlation with multiple variables from different layers, resulting in low path scores for trajectories that include it. In other words, these variables dynamically align with many other signals across the network, which make them more likely to be part of the most probable information paths. This is now explicitly stated in the Results (page 19, lines 473-483).

In the Discussion (pages 23, lines 568-580), we further clarify that, although MOCA is not intended as a standalone diagnostic tool, its centrality in our analysis highlights its utility as an integrative cognitive measure responsive to multimodal perturbations. Regarding the risk factor variables (HMSCORE, AXDIARRH, AXENERGY, AXDPMOOD, AXCRYING), their frequent appearance across paths is consistent with their behavioral and psychosocial nature, which positions them as sensitive mediators between physiological changes and cognitive symptoms. We have expanded the Discussion accordingly (pages 23, lines 568-580).

5. In the path analysis, one phenotype may link to multiple other phenotypes, could the authors add a SI table to show these results? It may be helpful to understand the AD complexity.

We appreciate this suggestion and agree that it is important to highlight the interdependencies between phenotypic variables. In response, we have included in the SI a set of matrices that report the number of connections between nodes within the phenotypic layer for each path analysis (Supplementary Section: Connectivity Between Phenotypic Variables in Path Analysis). These matrices provide detailed insight into the connectivity structure between cognitive and clinical phenotypes as derived from our dynamic pathway analysis. We have also added a reference to this supplementary figure in the main text (page 14, lines 394-397) to guide the reader.

Minor issues:

1. Line 354: “To identify the causal logic backbone of the network…” It may be too strong because the Boolean simulations might not reflect causal cascades.

Totally agree, saying “identify the causal logic backbone” implies that Boolean simulations prove causality, when in fact they are more of an approximation or qualitative model that does not guarantee direct causality. The statement in page 12, line 361 has been softened (“To explore the logical structure underlying the network...”) to reflect that the simulations explore or model the logic of the system, without ensuring

that it is causal in the strict sense.

2. Do all samples from collected ADNI cohort have all variables in each layer? If not, how the author constructs the network by considering all variables, e.g., by computing inter-layer MI? These details should be clarified.

An explanatory paragraph has been included on page 5, lines 163-167 that:

- Clarifies that not all subjects have data in all layers.
- Explains how the mutual information is calculated only with the subjects that have data available for each pair of variables.
- Justifies this methodological decision to maximize the use of available data.

3. Inconsistent count of subjects: the methods say 1776 participants (888 MCI, 348 AD, 540 controls), but the Results mention 622 controls, 807 MCI, 533 AD.

The numbers on page 3, line 62-64 in the Methods were incorrect and have been corrected with the count in the Results, which were the correct count.

4. Some inaccurate claims:
line 79: “more than 30 genetic variants”,
abstract: “average cognitive phenotype”,
line 426, 546: should include p value for “significantly”

The inaccuracies have been corrected on the same line where they were indicated (lines 80-81, Abstract and 444-446, 621, respectively).

5. Sometimes “subjects with mild AD” are mentioned, elsewhere just “AD patients”. They are different, please keep consistent.

I have distinguished between the entire cohort and the AD patients group (other than the control and MCI). The modifications can be seen in lines 289, 291, 385, 390 and 410.

6. Fig 9-13: missing color bars.

By changing the image format to TIF, all color bars are now displayed correctly.

Have the authors made all data and (if applicable) computational code underlying the findings in their manuscript fully available?

The PLOS Data policy requires authors to make all data and code underlying the findings described in their manuscript fully available without restriction, with rare exception (please refer to the Data Availability Statement in the manuscript PDF file). The data and code should be provided as part of the manuscript or its supporting information, or deposited to a public repository. For example, in addition to summary statistics, the data points behind means, medians and variance measures should be available. If there are restrictions on publicly sharing data or code —e.g. participant privacy or use of data from a third party—those must be specified.

Reviewer #1: **No:** I could see the GitHub link with the networks generated for the paper. However, I was unable to find the relevant code. If it has been uploaded, an easier access to it would be better.

Reviewer #2: **No:** Code is currently not accessible.

As mentioned above, the code is now available in page 25, lines 668-670.
