# Supplementary material for: Multiscale networks in Alzheimer’s disease identify brain hypometabolism as central across biological scales
Source: PLoS Comput Biol. 2025 Oct 17;21(10):e1013583. doi: 10.1371/journal.pcbi.1013583 (PMC12548887; doi:10.1371/journal.pcbi.1013583)
Supplement: Attachment — Submitted filename: Response_to_Reviewers_auresp_2.pdf [file pcbi.1013583.s026.pdf]

Reviewer's Responses to Questions

Reviewer #2: The authors well addressed my concerns. Overall, the manuscript looks much improved both in clarity and content. There are some minor comments:

1. As FDG PET was found as a key path for AD, but it is still not clear how this node connects with other nodes in other layers. Particularly for FDG PET, please describe more about its upstream and downstream connections to nodes in other layers. A small schematic table/figure would help readers.

To address this point, we have expanded the Results section to include a detailed description of FDG PET upstream and downstream connections across layers (lines 479-495). To provide a comprehensive overview, we have also included in the Supplementary Information a table listing all FDG PET nodes and their significant connections with nodes from other layers (Supplementary Table S8).

2. The names of “control group” in the text or tables are not consistent: the names “normal control, health control or Control” should be standardized.

The names have been standardized to “control” (lines 95, 141, 296, 299)

3. Page 11 line 303: “A threshold was implemented to examine…” Please clarify the threshold explicitly.

We have now clarified in the manuscript that the threshold was defined by comparing each pair’s mutual information value against a surrogate distribution obtained from random permutations of the data. Edges were retained only if the associated p-value was below 0.05 (lines 312-313)

4. Please improve Figures 6-14 to make the labels much clearly to readers.

To improve clarity, we have modified Figures 6–14 by labeling nodes numerically instead of using full variable names (which are lenghtly), which made the figures crowded, and we have increased their size. The full description of each variable and its corresponding numeric label is now provided in Supplementary Tables S1–S6, allowing readers to easily identify the nodes while keeping the figures clear and legible.

5. Glad to see the codes have been uploaded. For transparency and reproducibility, please deposit de-identified source data in github as well.

Unfortunately, we are not authorized to distribute ADNI data directly, as explicitly stated in the Data Use Agreement: “I understand that I am not allowed to distribute ADNI data outside of my team, only the ADNI study website operated by LONI-IDA can distribute data.”

To ensure transparency and reproducibility, we have updated the README file of our GitHub repository to include a link to the ADNI data portal, where researchers can apply for access. Once the data have been obtained, the code we provide can be run without modification to reproduce all analyses (lines 693-695).

In addition, we have added a new paragraph to the acknowledgments to comply with ADNI policies (lines 702-722).

Have the authors made all data and (if applicable) computational code underlying the findings in their manuscript fully available?

The PLOS Data policy requires authors to make all data and code underlying the findings described in their manuscript fully available without restriction, with rare exception (please refer to the Data Availability Statement in the manuscript PDF file). The data and code should be provided as part of the manuscript or its supporting information, or deposited to a public repository. For example, in addition to summary statistics, the data points behind means, medians and variance measures should be available. If there are restrictions on publicly sharing data or code —e.g. participant privacy or use of data from a third party—those must be specified.

Reviewer #2: **No**: Source data for the codes are not shown.

To ensure transparency and reproducibility, we have updated the README file of our GitHub repository to include a link to the ADNI data portal, where researchers can apply for access (lines 693-695).
