# Supplementary material for: Multiscale networks in Alzheimer’s disease identify brain hypometabolism as central across biological scales
Source: PLoS Comput Biol. 2025 Oct 17;21(10):e1013583. doi: 10.1371/journal.pcbi.1013583 (PMC12548887; doi:10.1371/journal.pcbi.1013583)
Supplement: Attachment — Submitted filename: review_PCOMPBIOL-D-24-01994.docx [file pcbi.1013583.s024.docx]

1. **What are the main claims of the paper and how significant are they for the discipline?**

The paper suggests that Alzheimer’s disease (AD) can be better understood using a systems biology approach that integrates genomic, molecular, and clinical data through multilayer network analysis. This approach highlights key connections between biological features and clinical symptoms, offering a comprehensive framework that could improve diagnostics and personalized treatments.

2. **Are these claims novel? If not, which published articles weaken the claims of originality of this one?**

While the approach of using systems biology and network analysis is innovative, it is not entirely novel. However, the integration of diverse biological scales (genomics, imaging, risk factors, etc.) could be considered a more novel aspect of this research.

3. **Are the claims properly placed in the context of the previous literature? Have the authors treated the literature fairly?**

The claims are supported by existing literature, with references to studies on brain hypometabolism and cardiovascular history in AD.

4. **Do the data and analyses fully support the claims? If not, what other evidence is required?**

The data support the claims, particularly in linking biological pathways to AD features. However, the cross-sectional design and single cohort (ADNI) limit causal conclusions. Longitudinal data and validation in other cohorts could further strengthen the findings.

5. **Would additional work improve the paper? How much better would the paper be if this work were performed and how difficult would it be to do this work?**

Additional work, such as using longitudinal data, larger sample sizes, and validation in other cohorts, would strengthen the paper. However, obtaining longitudinal data could be challenging due to the need for extended follow-up and more resources.

6. **PLOS Computational Biology encourages authors to publish detailed protocols and algorithms as supporting information online. Do any particular methods used in the manuscript warrant such treatment? If a protocol is already provided, for example for a randomized controlled trial, are there any important deviations from it? If so, have the authors explained adequately why the deviations occurred?**

The GitHub link to the networks generated is given. However, I had trouble finding the relevant code for this paper.

7. **Are original data deposited in appropriate repositories and accession/version numbers provided for genes, proteins, mutants, diseases, etc.?**

The manuscript does not explicitly mention whether the original data have been deposited in public repositories. The GitHub link is given along with the networks generated for the paper. However, I had trouble finding the relevant code for this paper.

8. **Has the author-generated code that underpins the findings been made publicly available?**

The GitHub link to the networks generated is given. However, I had trouble finding the relevant code for this paper.

9. **Does the study conform to any relevant guidelines such as CONSORT, MIAME, QUORUM, STROBE, and the Fort Lauderdale agreement?**

The manuscript does not explicitly state adherence to CONSORT, MIAME, QUORUM, STROBE, or the Fort Lauderdale agreement. However, it details its methodology, data sources, and ethical considerations. Contacting the authors could confirm this.

10. **Are details of the methodology sufficient to allow the experiments to be reproduced?**

In my opinion, the manuscript provides methodological details for reproducibility, including data from ADNI, network construction methods, statistical analyses, and GitHub-hosted code. However, full replication may require additional details on preprocessing, software versions, and parameter settings.

11. **Is any software created by the authors freely available?**

I had trouble finding the GitHub link. The only links provided in the article include the networks.

12. **Is the manuscript well organized and written clearly enough to be accessible to non-specialists?**

The manuscript is well-structured and informative.

13. **Does the paper use standardized scientific nomenclature and abbreviations? If not, are these explained at the first usage?**

Yes, the paper uses standardized scientific nomenclature and abbreviations. Moreover, the supplementary file contains all the abbreviations relevant to the study. However, terms such as “Aβ,” “PET,” and “MRI” are widely recognized in the field and clarifying these abbreviations upon their first use would improve understanding, especially for readers unfamiliar with the topic.
